# In Silico Discovery of Stapled Peptide Inhibitor Targeting the Nur77‐PPARγ Interaction and Its Anti‐Breast‐Cancer Efficacy

**DOI:** 10.1002/advs.202308435

**Published:** 2024-04-29

**Authors:** Huiting Bian, Xiaohui Liang, Dong Lu, Jiayi Lin, Xinchen Lu, Jinmei Jin, Lijun Zhang, Ye Wu, Hongzhuan Chen, Weidong Zhang, Xin Luan

**Affiliations:** ^1^ Shanghai Frontiers Science Center of TCM Chemical Biology Institute of Interdisciplinary Integrative Medicine Research and Shuguang Hospital Shanghai University of Traditional Chinese Medicine Shanghai 201203 China; ^2^ School of Pharmacy Fudan University Shanghai 201203 China; ^3^ School of Pharmacy Second Military Medical University Shanghai 200433 China; ^4^ Institute of Medicinal Plant Development Chinese Academy of Medical Science & Peking Union Medical College Beijing 100193 China

**Keywords:** breast cancer, lipid metabolism, Nur77‐PPARγ protein‐protein interaction, stapled peptide

## Abstract

The binding of peroxisome proliferator‐activated receptor γ (PPARγ) to the orphan nuclear receptor Nur77 facilitates the ubiquitination and degradation of Nur77, and leads to aberrant fatty acid uptake for breast cancer progression. Because of its crucial role in clinical prognosis, the interaction between Nur77 and PPARγ is an attractive target for anti‐breast‐cancer therapy. However, developing an inhibitor of the Nur77‐PPARγ interaction poses a technical challenge due to the absence of the crystal structure of PPARγ and its corresponding interactive model with Nur77. Here, ST‐CY14, a stapled peptide, is identified as a potent modulator of Nur77 with a *K*_D_ value of 3.247 × 10^−8^ M by in silico analysis, rational design, and structural modification. ST‐CY14 effectively increases Nur77 protein levels by blocking the Nur77‐PPARγ interaction, thereby inhibiting lipid metabolism in breast tumor cells. Notably, ST‐CY14 significantly suppresses breast cancer growth and bone metastasis in mice. The findings demonstrate the feasibility of exploiting directly Nur77‐PPARγ interaction in breast cancer, and generate what to the best knowledge is the first direct inhibitor of the Nur77‐PPARγ interaction available for impeding fatty acid uptake and therapeutic development.

## Graphical abstract

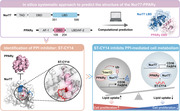

## 1 Introduction

Aberrant lipid metabolism plays key regulatory roles in the proliferation, aggressiveness, and metastasis of certain malignancies, especially breast cancer.^[^ ^1^, ^2^ ^]^ As the most diagnosed cancer with the leading mortality among women worldwide, breast cancer progression is often related to the unique mammary microenvironment, which is characterized by abundant lipocyte infiltration.^[^ ^3^, ^4^, ^5^ ^]^ Lipocytes provide a predominant source for energy metabolism and biomembrane synthesis, and are conducive to breast cancer growth and metastasis.^[^ ^5^ ^]^ Nur77 (also known as TR3 or NGFI‐B) has been demonstrated to be a potent tumor‐suppressive regulator because of its inhibitory function on lipid metabolism by blocking the transcription of the cluster of differentiation 36 (CD36) and fatty acid binding protein 4 (FABP4), the main proteins regulating fatty acid (FA) uptake.^[^ ^6^, ^7^, ^8^ ^]^ However, peroxisome proliferator‐activated receptor γ (PPARγ)‐induced Nur77 degradation by recruiting the ubiquitin ligase (E3) Trim13 prevents the positive regulation of Nur77, which promotes aberrant lipid uptake in breast cancer.^[^ ^6^ ^]^ Therefore, the interaction between Nur77 and PPARγ could provide a therapeutic target to inhibit lipid metabolism in breast cancer.

Nur77 contains an N‐terminal transcription‐activating domain, a DNA‐binding domain (DBD), and a ligand‐binding domain (LBD).^[^ ^9^ ^]^ Among them, the Nur77 LBD is responsible for PPARγ DBD binding. A previous study showed that the small molecule cytosporone B (Csn‐B) could indirectly impede the Nur77‐PPARγ interaction by promoting Nur77 homodimer formation, which in turn led to steric hindrance to prevent PPARγ binding.^[^ ^6^ ^]^ Given the pivotal role of mono Nur77 in breast cancer, directly inhibiting the Nur77‐PPARγ interaction would be extremely valuable for promoting mono Nur77 accumulation and binding to transcriptional corepressors to downregulate CD36 and FABP4 expression. Unfortunately, the unreported structural information currently available for the Nur77‐PPARγ complex has made the development of inhibitors directly targeting the Nur77‐PPARγ interaction extremely challenging.

**Scheme 1:**
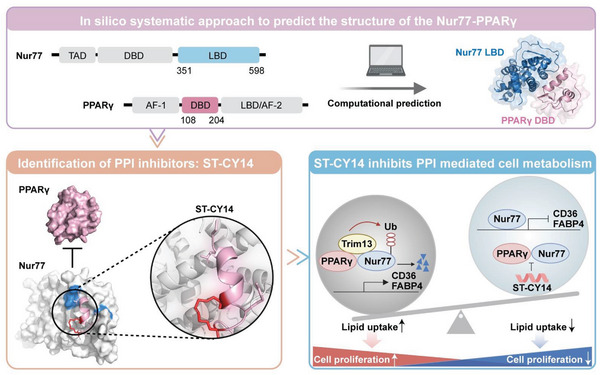
In silico approaches and chemical modifications are utilized to discover the first peptide inhibitor targeting the Nur77‐PPARγ interaction in the absence of cocrystal structures for breast cancer therapy.

Compared to small molecule inhibitors, peptides, the native binding motifs of proteins, have shown more promise as novel drug candidates for blocking protein–protein interactions (PPIs), especially flat (often exposed to solvent) and large (≈1500–3700 Å^2^) PPIs.^[^ ^10^ ^]^ Additionally, advanced computational approaches, including AlphaFold2, molecular docking, and molecular dynamics (MD) simulation, have been proposed to facilitate the discovery of critical binding epitopes and the design of peptide inhibitors for those PPIs without cocrystal structures.^[^ ^11^, ^12^, ^13^ ^]^ Here, to discover the first direct inhibitor of the Nur77‐PPARγ interaction, the aforementioned in silico approaches were systematically adopted to predict the highly plausible and stable binding conformation of the Nur77‐PPARγ complex, followed by peptide rational design, binding affinity evaluation, and structural stapling modification. Encouragingly, a potent stapled peptide modulator, ST‐CY14, bound to Nur77 with a *K*_D_ value of 3.247 × 10^−8^ M. By using ST‐ CY14 as a chemical probe, we found that directly blocking the Nur77‐PPARγ interaction promoted the accumulation of Nur77 and inhibited lipid absorption in breast tumor cells. Furthermore, animal experiments also validated the therapeutic potential of ST‐CY14 to inhibit breast cancer tumor growth and bone metastasis. Our work provided crucial insights into the Nur77‐PPARγ PPI and a new strategy for the development of anti‐breast‐cancer peptides targeting lipid metabolism (**Scheme** **1**).

## 2 Results and Discussion

### 2.1 Nur77 Inhibits Breast Cancer Proliferation, Lipid absorption, and Progression

**Figure 1:**
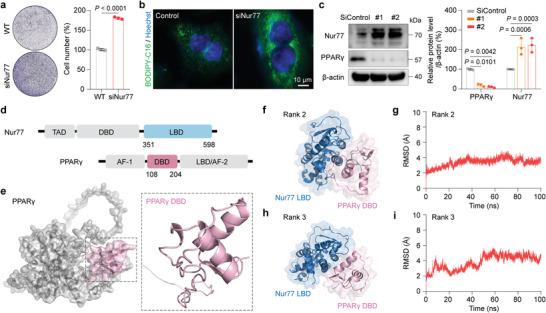
The function of Nur77 in breast tumor cells and an in silico systematic approach to predict the structure of the Nur77‐PPARγ heterodimer. a) Cell proliferation assays of Nur77‐knockdown MCF7 cells. The quantified data from different experiments were presented as the mean ± SD, and the *P* values were calculated using a two‐tailed *t‐*test. *n*  =  3 biological replicates. b) Enhanced lipid uptake capabilities were observed in Nur77‐knockdown MCF7 cells. c) Western blotting was used to determine the protein levels of Nur77 and PPARγ after knocking down PPARγ in MCF7 cells (PPARγ siRNAs are represented by #1 and #2). The quantified data from different experiments were presented as the mean ± SD. The *P* values were calculated by one‐way ANOVA. *n* = 3 biological replicates. d) Schematic representations of Nur77 and PPARγ. e) The structure of the PPARγ DBD predicted by AlphaFold2. f) Predicted binding conformation of the Nur77 LBD‐PPARγ DBD heterodimer (rank 2). g) The Cα RMSD for the rank 2 conformation. h) Predicted binding conformation of the Nur77 LBD‐PPARγ DBD heterodimer (rank 3). i) The Cα RMSD for the rank 3 conformation.

To define the antitumor role of Nur77 in breast cancer, we knocked down Nur77 in human breast cancer MCF7 cells using a Nur77 siRNA (siNur77). Nur77 knockdown significantly promoted the clonal proliferation of MCF7 cells (**Figure** **1**a). In addition, there were more long‐chain FAs in Nur77‐knockdown MCF7 cells than in wild‐type (WT) MCF7 cells (Figure 1b), implicating the suppressive role of Nur77 in breast tumor cell proliferation and lipid uptake. Overall survival analysis also indicated that Nur77 had a positive impact on the overall survival time of postoperative patients with breast cancer, while patients with higher levels of PPARγ expression had poorer prognoses (Figures S1, Supporting Information).^[^ ^6^ ^]^ According to the opposing roles of Nur77 and PPARγ in the clinical prognosis of breast cancer patients, we further found that the negative regulation of Nur77 protein expression by PPARγ was observed in PPARγ‐knockdown MCF7 cells (Figure 1c). These findings strongly support the notion that Nur77 and PPARγ are consistently linked to the clinical outcome of breast cancer, and that increasing Nur77 levels by blocking the Nur77‐PPARγ interaction holds promise for anti‐breast‐cancer strategies.

### 2.2 In Silico Systematic Approach to Predict the Structure of the Nur77‐PPARγ Heterodimer

To identify potent peptide inhibitors of the Nur77‐PPARγ interaction, we first determined the hot spots for Nur77‐PPARγ binding based on the Nur77 LBD spanning amino acids 351–598 (PDB: 6KZ5) and the PPARγ DBD (residues 108–204) (Figure 1d), which have been previously reported as interaction domains.^[^ ^6^ ^]^ We utilized AlphaFold2^[^ ^14^ ^]^ to predict the structure of PPARγ. The structure of the PPARγ DBD predicted by AlphaFold2 had a very high per‐residue confidence score (pLDDT >90) (Figure 1e; Figure S2a, Supporting Information). Both AlphaFold2 and Molecular Operating Environment (MOE)^[^ ^15^ ^]^ software were further employed to predict the binding conformation of the Nur77 LBD‐PPARγ DBD heterodimer. The predicted binding conformations of AlphaFold2 and the MOE (top 3 conformations, the ranking scores were shown in Figure S2b, Supporting Information) were evaluated using MD simulations by GROMACS.^[^ ^16^ ^]^ Among them, the conformation predicted by AlphaFold2 was found to be unstable (Figure S2c, Supporting Information). Figure 1f–i demonstrated that the structures ranked 2 and 3 predicted by MOE showed conformational stability according to the Cα root mean square deviations (RMSDs), while the structure ranked 1 exhibited instability, as evidenced by fluctuating RMSD values (Figure S2d, Supporting Information). Thus, the rank 2 and rank 3 binding conformations of the Nur77‐PPARγ complex were chosen to design peptide inhibitors.

### 2.3 Identification of Inhibitors of the Nur77‐PPARγ Interaction

Hotspot residues located on the protein‐protein binding surfaces are commonly exploited for the design of peptide inhibitors.^[^ ^17^ ^]^ According to the rank 2 conformation of the Nur77‐PPARγ complex (Figure S3a,b, Supporting Information), both a flexible loop of PPARγ (residues Ile167‐Leu178) named IL12 and an α‐helix of PPARγ (residues Ser186‐Val201) named SV16 bind one side of the Nur77 LBD, which indicates that IL12 and SV16 could be identified as potential peptide candidates. Similarly, another α‐helix of PPARγ (residues Cys156‐Leu169) named CL14 was obtained from the rank 3 binding conformation of the Nur77‐PPARγ complex (Figure S3c,d, Supporting Information). MD simulations revealed the stable binding of SV16 (Figure S3e, Supporting Information) and CL14 (Figure S3f, Supporting Information) to Nur77, whereas the binding between IL12 and Nur77 was found to be unstable (Figure S3g, Supporting Information). Therefore, SV16 and CL14 served as the starting points for our further design of peptide inhibitors.

**Figure 2:**
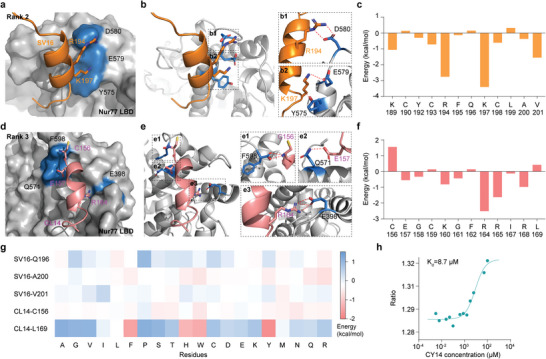
Identification of inhibitors targeting the Nur77‐PPARγ interaction. a) The interaction between SV16 and Nur77 was depicted in both cartoon and stick forms, with SV16 colored orange and the Nur77 LBD colored blue. Interaction residues were labeled. b) The detailed interaction between SV16 and Nur77 was revealed, with the red dashed line representing the intermolecular bond between them. c) The binding free energy decomposition of SV16 and Nur77 was shown. d) The interaction between CL14 and Nur77 was depicted in both cartoon and stick forms, with CL14 colored pink and the Nur77 LBD colored blue. Interaction residues were labeled. e) The detailed interaction between CL14 and Nur77 was presented, with the red dashed line representing the intermolecular bond between them. f) The binding free energy decomposition of CL14 and Nur77 was illustrated. g) The free energy changes after peptide point mutations. h) The binding affinity of CY14 with Nur77 was depicted using MST.

Key interaction residues between the Nur77 LBD and CL14 or SV16 were determined using molecular mechanics with generalized born and surface area solvation binding free energy decomposition. The analysis demonstrated that SV16 Arg194 and Lys197 contributed more to the binding free energy (**Figure** **2**c). SV16 Arg194 interacted with Nur77 Asp580 through a hydrogen bond, while SV16 Lys197 formed a hydrogen bond with Nur77 Glu579 and an H–π interaction with Tyr575 (Figure 2a,b). For CL14, Arg164 contributed the highest proportion of the binding free energy (Figure 2f) and could interact with Nur77 Glu398 by H‐bonds (Figure 2d,e). Additionally, the backbone of Cys156 and the side chain of Glu157 from CL14 engaged in hydrogen bonding interactions with Nur77 Phe598 and Gln571, respectively (Figure 2d,e).

To enhance the binding affinity of SV16 and CL14 with Nur77, amino acids near the binding surface that contributed less to the binding energy, as determined by energy decomposition, were mutated using Rosetta.^[^ ^18^ ^]^ Specifically, Gln196 and Ala200 of SV16 were subjected to mutation, while Cys156 and Leu169 of CL14 were mutated. Moreover, given the low contribution of SV16 Val201 to the binding energy of Nur77 and the proximity of its binding surface, Val201 of SV16 was also mutated (Figure S4a, Supporting Information). The mutation scores were presented in Figure 2g, with a noticeable decrease in binding energy when CL14 Leu169 was mutated to Tyr (CY14), Phe (CF14), His (CH14), or Trp (CW14). Among the aforementioned mutated peptides, the binding free energy of CY14, in which Leu169 was mutated to Tyr, displayed the most significant change, with a decrease of 1.74 kcal mol^−1^. Consequently, variants of the peptides, including SV16, CL14, CY14, CF14, CH14, and CW14, were generated and synthesized using standard Fmoc solid‐phase peptide synthesis (SPPS) procedures. The binding affinity of these peptides to Nur77 was evaluated using microscale thermophoresis (MST). As depicted in Figure 2h, Figure S4b,c (Supporting Information), CY14, which resulted from the mutation of CL14, exhibited the highest affinity for Nur77 (*K*_d CY14_ = 8.70 × 10^−6 ^_M_, *K*_d SV16_ = 1.56 × 10^−4 ^_M_, *K*_d CL14_ = 2.72 × 10^−5 ^_M_). We speculate that certain mutations (CF14, CH14, and CW14) may result in the instability of the peptide conformation, thereby reducing its binding affinity (Figure S4f−h). Furthermore, MD analysis confirmed the stable binding of CY14 with Nur77 (Figure S4d, Supporting Information). Notably, as illustrated in Figure S4e (Supporting Information), the presence of a benzene ring in Tyr facilitated π–π interactions with Nur77 Phe395, and the hydrophilic hydroxyl group of the tyrosine benzene ring was exposed to solvents. As a result, the mutated Tyr residue exhibited improved energy and geometric compatibility with Nur77.

### 2.4 Structural Optimization of CY14

**Figure 3:**
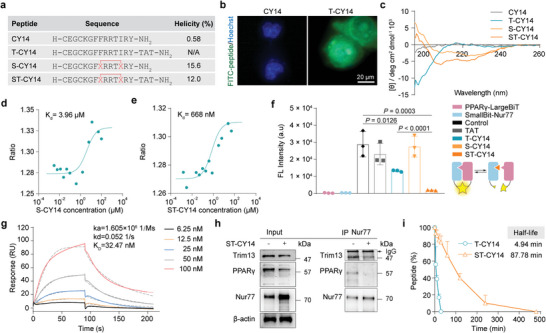
Structural optimization of CY14. a) Sequences and helicities of CY14, T‐CY14, S‐CY14, and ST‐CY14. X, S5 (*S*‐pentenyl alanine). H and NH_2_ in the peptide sequence represent the N‐terminal amino group and C‐terminal primary amide, respectively. b) The cell transmembrane abilities of CY14 and T‐CY14 were observed via confocal microscopy. c) CD spectra of T‐CY14 and ST‐CY14. d) The affinity of S‐CY14 for Nur77 was depicted using MST. e) The affinity of ST‐CY14 for Nur77 was depicted using MST. f) The blocking effect of ST‐CY14 on Nur77 and PPARγ was detected by NanoBit. The quantified data from different experiments were presented as the mean ± SD. The *P* values were calculated by one‐way ANOVA. *n*  =  3 biological replicates. g) The affinity of ST‐CY14 for Nur77‐LBD was determined using SPR. h) Immunoprecipitation of PPARγ was performed after treatment with ST‐CY14 (1 × 10^−5 ^_M_) in 293T cells. IP: Nur77, IB: PPARγ, Trim13. i) The proteolytic stability of T‐CY14 and ST‐CY14 in an α‐chymotrypsin solution.

To determine whether CY14 inhibits the Nur77‐PPARγ interaction in cells, we assessed its cell permeability by conjugating 5‐fluorescein isothiocyanate (5‐FITC) to CY14 (Table S2, Supporting Information). Unfortunately, CY14 cannot cross cell membranes to interact with intracellular target (**Figure** **3**a,b). Hence, we conjugated the transcription transactivating (TAT) sequence to the C‐terminus of CY14 (Figure 3a) using SPPS. FITC‐peptide conjugated with TAT (T‐CY14) exhibited significantly enhanced membrane permeability in MCF7 cells (Figure 3b). Additionally, although AlphaFold2 predicted that CY14 adopted an α‐helical conformation (Figure S4e, Supporting Information), neither CY14 nor T‐CY14 exhibited representative α‐helical characteristics in circular dichroism (CD) spectroscopy (Figure 3c). This difference may be attributed to the inherent tendency of linear peptides to lose their native topology in aqueous solution, which could explain the relatively low affinity of CY14 toward Nur77. As one of the most widely applied peptide‐stapling approaches for PPIs, all‐hydrocarbon stapling chemistry is an effective strategy for structurally stabilizing α‐helices and favoring protein binding.^[^ ^19^ ^]^ We synthesized stapled CY14 and T‐CY14 by incorporating S‐pentenyl‐alanine (*S*5) at the *i* and *i* + 4 positions (Phe163 & Ile167) along one face of the α‐helix exposed to the solvent surface (Figure S4j, Supporting Information), followed by ring‐closing olefin metathesis (Figures 3a; S4k,l, Supporting Information). Both stapled α‐helical peptides, S‐CY14 and ST‐CY14, displayed relatively high α‐helicities, with α‐helical degrees of 15.6% and 12.01%, respectively (Figure 3a,c). To verify that the staple did not impair the interaction between the peptide and Nur77, the affinities of S‐CY14 (*K*_d_ = 3.96 × 10^−6^ M) and ST‐CY14 (*K*_d_ = 6.68 × 10^−7^ M) with Nur77 were determined using MST (Figure 3d,e), demonstrating higher affinities than T‐CY14 (*K*_d_ = 4.18 × 10^−6^ M) (Figure S4i, Supporting Information).

To confirm the blocking function of ST‐CY14 on the Nur77‐PPARγ complex in cells, plasmid DNA constructs containing luciferase SmallBit fusions of Nur77 and luciferase LargeBit fusions of PPARγ were generated. These constructs were transiently expressed in HEK293T cells. The formation of the Nur77‐PPARγ complex contributes to luminescence due to the interaction between SmBit and LgBit upon the addition of the nanoluciferase substrate. The results showed that ST‐CY14 effectively inhibited the formation of the Nur77‐PPARγ complex, with an EC_50_ value of 3.15 × 10^−6^ M, in comparison to T‐CY14 (Figure S5, Supporting Information). Conversely, TAT and S‐CY14 had minimal effects on the luminescence. (Figure 3f). All these results demonstrated the power of the stapled peptide and the necessity of the cell‐penetrating sequence. Mutation of Nur77 at positions Gln571, Glu398, and Phe598 disrupted the interaction between Nur77 (Mut) and ST‐Y14 (Figure S4i, Supporting Information), providing additional evidence that ST‐CY14 primarily interacts with Nur77 through these three key residues. Surface plasmon resonance (SPR) analysis further confirmed that ST‐CY14 bound to Nur77 with a *K*_D_ value of 3.247 × 10^−8^ M (Figure 3g). Subsequently, we examined the ability of ST‐CY14 to interfere with the endogenous interaction between Nur77 and PPARγ in HEK293T cells. Decreased PPARγ expression was detected in the ST‐CY14‐treated group, indicating that ST‐CY14 inhibited the binding between Nur77 and PPARγ. Trim13 was reported to mediate the ubiquitination of Nur77.^[^ ^20^ ^]^ ST‐CY14 obviously reduced the expression of Trim13 and attenuated the PPARγ‐induced ubiquitination of endogenous Nur77 (Figure 3h). These data suggested that ST‐CY14 could bind to Nur77 and specifically inhibit the Nur77‐PPARγ interaction in cells. Owing to the preorganized stable α‐helix topology, ST‐CY14 exhibited significantly greater proteolytic stability than T‐CY14 (half‐life 4.94 min vs 87.78 min), representing a seventeen‐fold improvement (Figure 3i).

### 2.5 ST‐CY14 Inhibits the Nur77‐PPARγ‐Mediated Signaling Pathway

**Figure 4:**
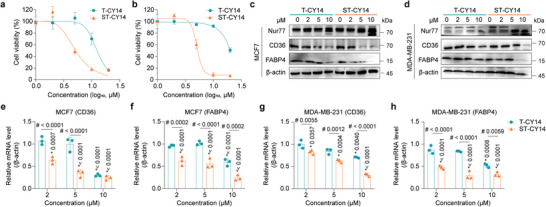
ST‐CY14 inhibits the Nur77‐PPARγ‐mediated signaling pathway. The cell viability of a) MCF7 and b) MDA‐MB‐231 cells after treatment with T‐CY14 or ST‐CY14 for 24 h. The effects of ST‐CY14 on increasing Nur77 protein levels and decreasing CD36 and FABP4 protein levels were more pronounced in c) MCF7 cells and d) MDA‐MB‐231 cells compared to those of T‐CY14. ST‐CY14 treatment inhibited the transcription of CD36 and FABP4 in e,f) MCF7 cells and g,h) MDA‐MB‐231 cells. The quantified data from different experiments were presented as the mean ± SD. The *P* values were calculated by two‐way ANOVA. *n*  =  3 biological replicates. * represents different concentrations of T‐CY14 or ST‐CY14 versus control group. # represents ST‐CY14 versus T‐CY14 at the same concentration.

We next investigated the cell viability of MCF7, MDA‐MB‐231, and MDA‐BoM‐1833 cells treated with ST‐CY14, T‐CY14, and Csn‐B, a reference control. ST‐CY14 at 5 × 10^−6 ^_M_ significantly attenuated breast tumor cell proliferation, and ST‐CY14 was more potent for breast tumor cells than T‐CY14 and Csn‐B (**Figure** **4**a,b: Figure S6, Supporting Information), which was in agreement with their respective binding affinities to Nur77. Moreover, normal breast cells (MCF‐10A) and Nur77‐knockdown breast tumor cells were less sensitive to ST‐CY14 (Figure S7, Supporting Information). In addition to its antiproliferative activity, ST‐CY14 exhibited a high solubility (>20 mg mL^−1^) in PBS, whereas the solubility of Csn‐B was significantly lower at ≈1 mg mL^−1^ (Figure S8, Supporting Information). These results highlight the advantage and potential of targeting the Nur77‐PPARγ interaction interface with a peptide inhibitor rather than a Nur77 agonist.

Blockade of the Nur77‐PPARγ interaction could increase Nur77 protein levels via decreased PPARγ‐induced ubiquitination.^[^ ^6^ ^]^ To this end, immunoblotting was employed and revealed that an increase in Nur77 expression in MCF7 and MDA‐MB‐231 cells following treatment with ST‐CY14 and T‐CY14 (Figure 4c,d). Moreover, ST‐CY14 inhibited the protein expression of CD36 and FABP4, which are regulated by Nur77, in MCF7 (Figure 4c) and MDA‐MB‐231 cells (Figure 4d). T‐CY14 also had some effect, but its efficacy was much lower (Figure 4c,d). Consistently, qPCR revealed that ST‐CY14 dose‐dependently inhibited CD36 and FABP4 expression at the transcriptional level in MCF7 (Figure 4e,f) and MDA‐MB‐231 cells (Figure 4g,h).

### 2.6 ST‐CY14 Inhibits Nur77‐PPARγ‐Mediated Cell Metabolism

**Figure 5:**
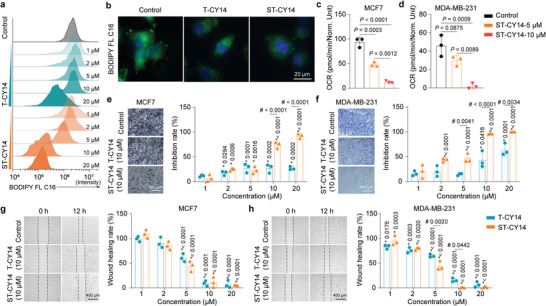
ST‐CY14 inhibits Nur77‐PPARγ‐mediated cell metabolism. a) Flow cytometry was used to compare the lipid uptake abilities of MCF7 cells after treatment with T‐CY14 or ST‐CY14. b) Confocal microscopy images were taken to observe lipid uptake in MCF7 cells after 1 × 10^−5^ M T‐CY14 or ST‐CY14 treatment. ST‐CY14 significantly inhibited mitochondrial aerobic respiration in c) MCF7 and d) MDA‐MB‐231 cells. The quantified data from different experiments were presented as the mean ± SD. The *P* values were calculated by one‐way ANOVA. *n*  =  3 biological replicates. Representative images and statistics of Transwell migration assays of e) MCF7 and f) MDA‐MB‐231 cells following treatment with T‐CY14 and ST‐CY14. Representative images and statistics of wound‐healing assays on g) MCF7 and h) MDA‐MB‐231 cells after treatment with T‐CY14 and ST‐CY14. For (e–h), the quantified data from different experiments were presented as the mean ± SD. The *P* values were calculated by two‐way ANOVA. *n*  =  3 biological replicates. * represents different concentrations of T‐CY14 or ST‐CY14 versus control group. # represents ST‐CY14 versus T‐CY14 at the same concentration.

Nur77 acts as a suppressor of CD36 and FABP4, which regulate lipid metabolism during lipid uptake and lipid decomposition. To determine whether direct blockade of the Nur77‐PPARγ interaction is involved in lipid metabolism, we used ST‐CY14 as a chemical probe to treat breast tumor cells. Examination of FA uptake by flow cytometry revealed that ST‐CY14 induced a dose‐dependent decrease in FA levels in MCF7 cells (**Figure** **5**a). Immunofluorescence‐based studies also showed that ST‐CY14 inhibited FA uptake in MCF7 cells (Figure 5b). FAs are the main substrates for mitochondrial energy metabolism; thus, we further evaluated the oxygen consumption rate (OCR), an indicator of mitochondrial respiration, in MCF7 and MDA‐MB‐231 cells following ST‐CY14 treatment. These results showed that the OCR was significantly reduced after ST‐CY14 treatment, indicating that ST‐CY14 reduced the rate of mitochondrial respiration in MCF7 (Figure 5c) and MDA‐MB‐231 cells (Figure 5d).

As the main component of the cell membrane and energy metabolism substrate, FAs are vital in cell migration and invasion in breast cancer metastasis. We wondered whether ST‐CY14 might have inhibitory effects on the migration of breast tumor cells. Our transwell experiments showed that ST‐CY14 and T‐CY14 inhibited the transmigration of MCF7 (Figure 5e) and MDA‐MB‐231 (Figure 5f) cells. ST‐CY14 and T‐CY14 also suppressed the scratch healing of both breast tumor cell lines (Figure 5g,h). Taken together, these results suggest that direct blockade of the Nur77‐PPARγ interaction inhibits the metabolism, migration, and invasion of breast tumor cells.

### 2.7 ST‐CY14 Suppresses Tumor Growth and Bone Metastasis of Breast Cancer in Mice

**Figure 6:**
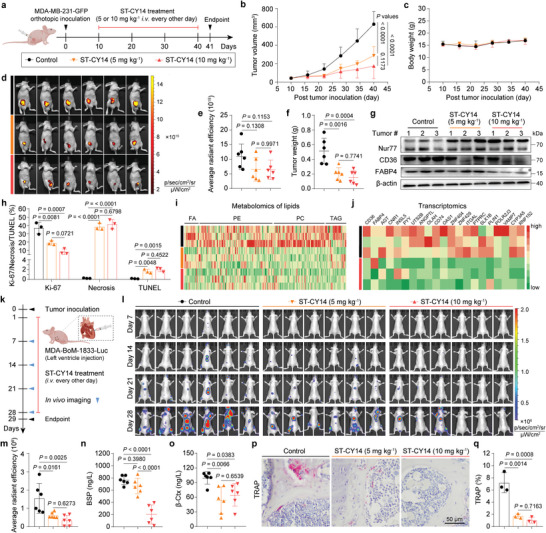
ST‐CY14 suppresses tumor growth and bone metastasis of breast cancer in mice. a) Flow diagram of the treatment regimen in the MDA‐MB‐231‐GFP xenograft model. b) Tumor volume during treatment (n = 6). c) Time‐course of body weight (n = 6). d) In vivo tumor imaging before sacrifice and e) accumulated optical density measurement. f) Tumor weight. g) Western blot analysis showing that ST‐CY14 treatment inhibits Nur77 and its downstream signaling pathway‐related target genes CD36 and FABP4 in vivo. h) Image‐based quantitative results of Ki‐67, H&E, and TUNEL staining of tumor samples (n = 3). i) Heatmap images showing a significant reduction in the levels of diverse lipid constituents within the group treated with ST‐CY14. j) Heatmap images of the nearly twenty downregulated genes. k) Flow diagram of the treatment regimen in bone metastatic breast cancer model. l) In vivo imaging of mice every week. m) Bone metastasis tumor accumulated optical density measurement on day 28. n) BSP and o) β‐CTx levels in mouse blood detected through ELISA analysis. Immunofluorescence staining images of the p) TRAP ratio in the mouse femoral head and q) relative statistics. For (b,e,f,h,m–o, and q), the quantified data from different experiments were presented as the mean ± SD. The *P* values were calculated by one‐way ANOVA.

Following the above encouraging results observed for ST‐CY14 in cells, we evaluated the antitumor efficacy of ST‐CY14 in an MDA‐MB‐231‐GFP xenograft model (**Figure** 6a). ST‐CY14 suppressed tumor growth in a dose‐dependent manner by 60.18% and 69.73% tumor growth inhibition values at the end of the thirty‐day treatment after receiving 5  and 10 mg kg^−1^ doses, respectively, once every two days via the tail vein (Figure 6b), without significant body weight loss (Figure 6c) or other signs of organ toxicity (Figure S9, Supporting Information) in all treated mice. Moreover, bioluminescence imaging and tumor weight also indicated that ST‐CY14 inhibited tumor progression in mice (Figure 6df). Consistent with the in vitro results, ST‐CY14 effectively increased Nur77 protein levels, and decreased CD36 and FABP4 protein levels (Figure 6g) in MDA‐MB‐231‐GFP tumors. Pathological and immunohistochemical analyses of tumor sections showed that, compared with the control group, ST‐CY14 led to reduced tumor cell proliferation, increased necrosis area and apoptosis (Figures 6h; S10, Supporting Information). Considering the regulatory effect of Nur77 on lipid metabolism, lipid metabolome analysis was applied to detect the lipid content in tumor tissue. The relative abundance of various lipid content, such as FA, phosphatidyl choline (PC), triacylglycerol (TAG), and phosphatidyl ethanolamine (PE), decreased in the ST‐CY14‐treated group compared with the control group (Figure 6i; Dataset S1, Supporting Information). To further delineate the molecular mechanisms of ST‐CY14, transcriptome analysis of tumor tissue was performed. Differentially expressed gene enrichment analysis identified nearly twenty downregulated genes, including cell adhesion‐related molecules (PTPRC and INGAL), FA synthesis‐related molecules (OLAH), PPAR signaling pathway‐related molecules (CD36, ANGPTL4, and PLIN1), cholesterol metabolism‐related molecules (CD36 and CYP3A5), and lipolysis‐related molecules (FABP4 and PLIN1), in the ST‐CY14 treatment group (Figure 6j; Dataset S2, Supporting Information). Collectively, ST‐CY14 effectively suppresses Nur77‐PPARγ‐mediated breast cancer progression.

The bone is the most common site of breast cancer metastasis, accounting for ≈65 to 75% of all metastatic breast cancer patients. In particular, when metastasis occurs, the 5‐year survival rate of breast cancer patients is as low as 10%.^[^ ^21^ ^]^ In addition, enhanced lipid levels in the bone marrow benefit FA metabolism in breast cancer.^[^ ^22^ ^]^ Thus, a bone metastatic breast cancer model was used to evaluate the anti‐metastatic efficacy of ST‐CY14. A bone metastasis model was constructed by injecting 1 × 10^5^ MDA‐BoM‐1833 cells into the left cardiac ventricle of mice. Bone metastatic tumor progression was monitored by in vivo bioluminescence imaging (days 7, 14, 21, and 28) (Figure 6k). As shown in Figure 6l,m, significant bone metastasis was observed in the control group after the 28th day, while the fluorescence intensities of the ST‐CY14 treated groups were lower, suggesting that ST‐CY14 inhibited the metastasis of tumor cells. Tumor cells can secrete factors into the bone microenvironment, including IL‐6 and PTH‐related peptide, which activate osteoclasts when they are deposited in the bone marrow.^[^ ^23^ ^]^ Beta C‐terminal cross‐linked telopeptides of type I collage (β‐CTx) and Bonesialo protein (BSP) are considered to be markers of osteoclasts. Lower levels of BSP (Figure 6n) and β‐CTx (Figure 6o) were detected in the ST‐CY14‐treated group than in the control group, indicating that ST‐CY14 inhibited bone metastasis and the formation of osteoclasts in the bone marrow. None of the groups demonstrated notable changes in H&E staining of major organs, indicating satisfactory safety profiles (Figure S11, Supporting Information). Immunohistochemical analysis also showed that ST‐CY14 treatment led to a decrease in the level of tartrate‐resistant acid phosphatase (TRAP), the signature enzyme of osteoclasts (Figure 6p,q). Notably, the efficacy of ST‐CY14 in inhibiting breast cancer bone metastasis was superior to that of Csn‐B (Figure S12, Supporting Information). Taken together, these results demonstrated that ST‐CY14 is a promising lead compound to inhibit breast cancer bone metastasis.

## 3 Conclusion

The highly metastatic nature of breast cancer results in poor prognosis and heightened mortality rates. Adjacent lipocytes establish a lipid‐rich microenvironment, providing breast cancer cells with important substrates for proliferation, migration, and invasion. Thus, targeting lipid metabolism in breast cancer represents a promising direction for more effective therapeutic strategies. Extensive evidence has shown that Nur77 is a critical suppressor of lipid metabolism in breast cancer, and Nur77 deficiency often indicates a worse prognosis.^[^ ^6^ ^]^ However, the activated Nur77‐PPARγ signaling pathway plays an antagonistic role in the Nur77‐mediated process, and identifying direct inhibitors of the Nur77‐PPARγ interaction without a cocrystal structure is challenging.

Here, based on the optimal scaffolds of epitope peptides and advanced in silico approaches, we developed ST‐CY14 as a high‐potency PPI peptide inhibitor that effectively blocks the Nur77‐PPARγ interaction both in vitro and in vivo, which not only validates the feasibility of exploiting the Nur77‐PPARγ interaction as a target of breast cancer for drug discovery, but also provides an effective paradigm for computer‐aided drug design in PPI‐peptide‐inhibitor research. In the present study, our data demonstrated that the binding of ST‐CY14 to the Nur77 LBD could protect Nur77 from ubiquitination induced by PPARγ, leading to the accumulation of Nur77 and low levels of CD36 and FABP4. More importantly, due to its ability to reduce lipid metabolism, ST‐CY14 exhibited pronounced inhibitory efficacy on tumor growth and bone metastasis of breast cancer in mice.

In summary, we developed a potent and selective peptide inhibitor of Nur77‐PPARγ PPI, ST‐CY14, with in vivo antitumor activities and no toxicity. The structural scaffold and binding mode of ST‐CY14 make it a promising lead compound for further development in breast cancer treatment, and will facilitate the further design and modification of inhibitors in the Nur77‐PPARγ interface.

## 4 Experimental Section

### Materials, Cell Culture, and Animal Models

Rink Amide MBHA resin (0.30 mmol g^−1^ loading) was purchased from Tianjin Nankai Hecheng Science & Technology Co., Ltd.; All the other chemical reagents and solvents used were purchased from Adamas‐beta, GL Biotech, CSBio (Shanghai), Energy Chemical, and Sinopharm Chemical Reagent Co., Ltd, and were used without further purification.

The HEK293T, MCF7, and MDA‐MB‐231 cell lines were obtained from the Cell Bank of the Chinese Academy of Sciences (Shanghai, China). The MDA‐MB‐231 cell line was labeled with EGFP and luciferase by Xinzhou. Additionally, the MDA‐BoM‐1833 cell line was provided by Professor Yu‐Dong Zhou from the University of Mississippi and labeled with a luciferase tag in the lab. All cells were cultured in Dulbecco's modified Eagle's medium (DMEM) medium supplemented with 10% (v/v) fetal bovine serum and antibiotics (100 mg mL^−1^ streptomycin and 100 units mL^−1^ penicillin), and maintained at 37 °C with 5% CO_2_.

Female BALB/c mice (4–5 weeks old, 16–18 g) and BALB/c mice (7–8 weeks old, 18–20 g) were provided by Shanghai Slake Experimental Animal Co., Ltd., and housed under specific pathogen‐free conditions for use in orthotopic xenograft tumor model experiments and bone metastasis tumor model experiments. The animal experiment was approved by the ethical committee of Shanghai University of Traditional Chinese Medicine (approval number: PZSHUTCM220627036).

### Molecular Dynamics Simulation

The MD simulations were performed using GROMACS^[^ ^16^ ^]^ The Amber99SB‐ILDN force field and TIP3P water model were set to generate the topology of the protein complex. And then a dodecahedron box was created to place the protein at the center with a minimum distance of 1.2 nm between the protein and the box edges. The system was subjected to energy minimization in vacuo using the steepest descent algorithm to remove any steric clashes or bad contacts. To mimic the physiological environment, the protein was solvated using a TIP3P water model, and the ions were added to neutralize the system. Energy minimization was performed again, and the system was equilibrated at NVT and NPT conditions to maintain constant temperature (310 K) and pressure (1 bar). Finally, the production simulation was carried out for a total of 100–150 ns with a time step of 2 fs.

### Transient Transfection of siRNA

Cells were seeded 24 h prior to transfection. Transfection was performed with Transfect‐Mate (GenePharma, G04009) according to the manufacturer's protocols. To transfect siRNA into cells seeded on 6 well cell culture plates, 150 pmol of siRNA was mixed with 8 µL Transfect‐Mate, incubated at room temperature for 15 min, and then added to the cells. The medium was changed to DMEM after 6 h of culture, and the cells were incubated at 37 °C in a 5% CO_2_ incubator. 48 h later, the siRNA was successfully expressed and could be used for further experiments. The siRNAs used were listed in Table S1 (Supporting Information).

### Colony Formation Assay

The colony formation assay was used to evaluate the proliferative ability of the MCF‐7 cells. Transfected cells in the logarithmic growth phase were seeded into 6‐well plates. After siRNA transfection the next day and 1‐week incubation subsequently, visible colonies formed. Then, the cells were fixed and stained using methanol and Giemsa, respectively. Colonies were snaped under a microscope and counted at a wavelength of 590 nm.

### Synthesis of Peptides

Peptides were synthesized using SPPS on Rink Amide MBHA resin. The resin (1 mmol) was swelled with dichloromethane (DCM) and then deprotected twice using a deprotect solvent (N,N‐dimethylformamide, DMF, containing 20% piperidine and 0.1 mol L^−1^ Oxyma pure). The peptides were washed with DMF and DCM. A solution of Fmoc‐AA‐OH (1 mmol), N, N’‐diisopropylcarbodiimide (DIC) (1 mmol), and Oxyma pure (1 mmol) in N‐methylpyrrolidone (NMP) was added and reacted with the resin at 60 °C for 15 min. After the reaction, the resin was washed with DMF and DCM and deprotected for the next amino acid coupling. The 2 h coupling of Fmoc‐S5‐OH was carried out at 60 °C. After completion of all amino acid couplings, peptide cyclization was conducted using a 1 × 10^−2^ M solution of the Grubbs first‐generation ruthenium catalyst, benzylidene‐bis(tricyclohexylphosphine) dichlororuthenium, in dichloroethane. The peptide was cleaved from the resin using a cocktail of trifluoroacetic acid (TFA), triisopropylsilane, and H_2_O (95:2.5:2.5) for 2 h at room temperature.

### Fluorescent Labeling of Peptides

After elongation of the peptide from the resin according to a previous protocol, the Fmoc group was removed and the resin was treated with Fmoc‐Ahx‐OH (4 eq.), DIC (4 eq.), and Oxyma pure (4 eq.) in NMP for 15 min at 60 °C. Subsequently, the N‐terminal amino group was deprotected and reacted with 5‐FITC (2 eq.) and N,N‐diisopropylethylamine (2 eq.) in DMF (6 mL) at room temperature overnight, resulting in the incorporation of a fluorescein label.

### Analytical Methods

The detection wavelengths of the peptides were 214 and 254 nm. Solvent A: H_2_O (containing 0.1% TFA) and solvent B: acetonitrile (containing 0.1% TFA) were used as solvents in linear gradient mixtures. Peptide purification was performed by semi‐preparative HPLC using an XBridge Prep C_18_ column (19 × 250 mm, 10 µm particle size, flow rate of 20 mL min^−1^). Solvents A and B were the same as those in the peptide analysis procedure. The peptides were dissolved in H_2_O to a final concentration of 5 × 10^−5^ M. The purified peptide was detected using a mass spectrometer (6410 Triple Quad LC/MS, Agilent, USA) (Table S2, Supporting Information). The solvent was 80% methanol and 20% H_2_O (containing 0.1% formic acid). The CD spectra were obtained with a 1 mm quartz cuvette on a BRIGHT TIME Chirasca (Applied Photophysics, Britain) spectropolarimeter with a scan wavelength between 185–260 nm at 25 °C. The helicity of each peptide was calculated by the literature equation.^[^ ^24^ ^]^

### Observation of FITC‐peptide Translocation Using Confocal Microscopy

MCF7 cells (50 000 cells/well) were seeded in a 20 mm dish and treated with 1 × 10^−5^ M FITC‐labeled peptides for 1 h. After treatment, the cell nuclei were stained with Hoechst 33 342 (5 × 10^−5^ M) for 8 min. The membrane detection of peptides was performed using a Leica TCS SP8 confocal microscope. The FITC‐labeled peptide was excited at a wavelength of 488 nm, with an emission wavelength range of 505–530 nm, while the cell nuclei were excited at a wavelength of 350 nm, with an emission wavelength of 461 nm.

### Protein Expression and Purification

The LBD fragment of human Nur77 (residues 351–598), tagged with an N‐terminal His6‐tag, was inserted into the pET‐15b vector. The protein was expressed in *Escherichia coli* BL21(DE3) cells (Weidibio, EC1002S). The bacterial cultures were grown at 37 °C until they reached an optical density at 600 nm of 0.6. Then, 1 × 10^−4^ M IPTG (CAS: 367‐93‐1, Bidepharm, BD134860) was added to induce Nur77 (LBD) expression at 16 °C overnight. Subsequently, the bacteria were harvested. For the purification of Nur77 (LBD), the bacteria were lysed by sonication in a lysis buffer (5 × 10^−2^ M Tris‐HCl, pH 8.0, 0.5 _M_ NaCl, 5 × 10^−3^ M β‐mercaptoethanol, 10% glycerol, 1 × 10^−3^ M phenylmethane sulfonyl fluoride, 2 × 10^−2^ M imidazole). The lysate was centrifuged at 18 000 g for 30 min to remove cell debris. The supernatant was loaded onto a Ni^2+^ NTA‐agarose column (Qiagen). After the column was washed with lysis buffer, it was eluted with buffer containing 5 × 10^−2^ M Tris‐HCl (pH 8.0, 0.5 _M_ NaCl, 5 × 10^−3^ M β‐mercaptoethanol, 10% glycerol, and 0.25 m imidazole. The protein was then subjected to a HiLoad 16/60 Superdex 200 pg gel filtration column (Cytiva) and eluted with a buffer containing 2.5 × 10^−2^ M Tris‐HCl, pH 8.0, 0.2 _M_ NaCl, and 2 × 10^−3^ M Dithiothreitol. The fractions containing Nur77 (LBD) were collected and concentrated to 2 mg mL^−1^ using concentrators with a 10 kDa MWCO. Finally, the samples were flash‐frozen in liquid nitrogen and stored at −80 °C.

### Microscale Thermophoresis (MST)

The MST measure for the affinity of the peptide with purified Nur77 LBD protein was performed by Dianthus (Nano Temper). First, 1 × 10^−5^ M protein was labeled with fluorescent dye and incubated with variable concentrations (0.1/0.4/0.8/1.6/3.1/6.3/12.5/25/50/100 × 10^−6^ M) of peptides in PBST (containing 0.05% Tween‐20) (v/v, 10 µL: 10 µL). Incubated at room temperature away from light for 20 min, the mixture was loaded into the capillary and scanned. The MST measure for the affinity of the peptide with mutated Nur77‐EGFP was performed by Monolith NT.115 (Nano Temper). The HEK293T cell lysate was collected after transfection with the mutated Nur77‐EGFP plasmid for 48 h. The level of mutated Nur77‐EGFP was verified by the detection of fluorescence intensity. Different concentrations (0.1/0.4/0.8/1.6/3.1/6.3/12.5/25/50/100 × 10^−6^ M) of ST‐CY14 were mixed with the cell lysate (v/v, 5 µL:5 µL) and incubated for 20 min. The measurement was carried out at 40% MST power.

### Surface Plasmon Resonance (SPR)

ST‐CY14 with Nur77‐LBD was detected by SPR using the Biacore T200 system at 25 °C. Recombinant human Nur77‐LBD protein was immobilized on an activated carboxymethylated 5 sensor chip (GE Healthcare) using the amine coupling method. Gradient concentrations of EB were injected at a flow rate of 30 µL min^−1^ in a running buffer (1% DMSO in PBS). The results were analyzed with Biacore evaluation software (T200 version 2.0). The data were fitted to the 1:1 Langmuir binding model, and kinetic parameters were derived.

### Co‐Immunoprecipitation (Co‐IP)

HEK293T cells were seeded overnight in 10 cm culture dishes and transfected with the Nur77 and PPARγ plasmids for 48 h. After transfection, T‐CY14 and ST‐CY14 were incubated with the cells for 24 h. The cells were then collected and lysed in 500 µL precooled NP40 lysis buffer (Beyotime, P0013F), which contained 20x holoenzyme inhibitor and 50x phosphatase inhibitor. All the samples were prepared at a concentration of 1 mg mL^−1^, and 0.5 mg of the sample was subsequently incubated overnight at 4 °C with 2 µg of Nur77 antibody (Proteintech, 12235‐1‐AP). The samples were then incubated with Protein A/G PLUS‐Agarose (Santa Cruz, sc‐2003) for 4 h at 4 °C. The beads were collected through centrifugation and washed five times with NP40 lysis buffer. Finally, 5x loading buffer was added to the buffer containing the beads for Western blot analysis of PPARγ antibody (Proteintech, 16643‐1‐AP), Trim13 antibody (Abcam, ab234847), and β‐actin antibody (epizyme, LF201).

### Protease Stability

The peptides were dissolved in PBS (pH 7.4) to a final concentration of 1 × 10^−3^ M. α‐Chymotrypsin was dissolved in PBS (5 × 10^−2^ M, containing 2 × 10^−3^ M CaCl_2_, pH 7.4) to a final concentration of 0.5 ng µL^−1^. Subsequently, 100 µL peptides were incubated with α‐chymotrypsin (1 mL) at room temperature, and 100 µL of the digestion mixture was collected at different times (5/10/20/30/60/120/240/480 min) and added to a centrifuge tube containing 20 µL of 1 _M_ HCl to quench the enzymatic reaction. The solution of α‐Chymotrypsin peptide fragments was monitored by analytical HPLC to determine the fraction of protease degradation at 214 nm.

### Solubility Test

Mix 1 mg ST‐CY14 or Csn‐B (Shanghai Sunny Biotech) in 50 µL PBS buffer, and then transfer 40 µL the suspension to a 96‐well plate. The suspension was gradient diluted from 20 to 0. 07 8125 mg mL^−1^. OD values were measured at 600 nm on a Cytation 5 (Biotek, USA) plate reader. PBS was set as a blank control.

### Western Blot Analysis

MCF7 and MDA‐MB‐231 cells were treated with various concentrations of peptide. The cells were then collected and lysed in NP40 lysis buffer. Total protein was quantified using a BCA assay kit (Beyotime, P0010S). The proteins were separated by SDS‐PAGE and transferred onto a PVDF membrane. The PVDF membrane was blocked in TBST containing 5% non‐fat milk, and then incubated with primary antibodies overnight at 4 °C. Subsequently, membranes were incubated with horseradish peroxidase‐conjugated antirabbit or antimouse IgG secondary antibody for 1 h at room temperature. The membranes were analyzed by a Tanon‐5200 Multi Gel Imaging Analysis System (Tanon) and quantified by ImageJ software.

### NanoBiT Assay

HEK293T cells were seeded in a poly‐d‐lysine‐coated black 96‐well cell culture plate at 10 000 cells per well in 100 µL culture medium. The next day, the master mix of 100 ng receptor construct containing the PPARγ‐LargeBit and SmallBit‐Nur77 plasmids (w/w, 50 ng:50 ng) was diluted in Opti‐MEM Medium (Gibco, 11058‐021), then P3000 Reagent was added. Meanwhile, 0.15 µL Lipofectamine 3000 reagent (ThermoFisher, L3000015) was added to 5 µL Opti‐MEM Medium. Subsequently, diluted DNA was added to Lipofectamine 3000 reagent (V/V, 1:1) and incubated for 15 min at room temperature. A mixture containing the above plasmids and Lipofectamine 3000 was prepared and added to each well. After 24 h, the cells were treated with 1 × 10^−5^ M peptides (TAT, T‐CY14, S‐CY14, and ST‐CY14) or variable concentrations of ST‐CY‐14 (0.1/0.2/0.5/1/2/5/10/20/40 × 10^−6^ M) for 1 h. The culture medium was replaced with 90 µL Tyrode's solution (Solarbio, T1420). Then, 10 µL furimazine (5 × 10^−5^ M) was added to each well and the luminescence signal was measured every 86 s for 1 h at 37 °C.

### Cell Viability Assay

MCF7, MDA‐MB‐231, and MDA‐BoM‐1833 cells (5000 cells/well) were seeded in 96‐well plates and cultured overnight at 37°C and 5% CO_2_. The cells were then treated with fresh basic medium containing various concentrations (1/2/5/10/20 × 10^−6^ M) of compounds. After 24 h of incubation, the cells were incubated with a basic medium containing 10% Cell Counting Kit‐8 (Dojindo, CK‐04) for 2 h. Cell viability was evaluated by measuring the absorbance at 450 nm. The formula used was [(As‐Ab)/(Ac‐Ab)] × 100%, where as represents the absorbance of the drug‐treated group, Ac represents the absorbance of the control group treated with medium, and Ab represents the absorbance of the blank control containing only medium.

### Long‐chain Fatty Acid Uptake Assay

5×10^4^ MCF7 cells were seeded in confocal dishes and cultured overnight at 37 °C and 5% CO_2_. The cells were then incubated with a basic DMEM medium containing various concentrations of peptides (1/2/5/10/20 × 10^−6^ M for flow cytometry and 10 × 10^−6^ M for immunofluorescence) for 24 h. Afterward, 1 µL BODIPY FL C16 (1 × 10^−6^ M, Thermo Fisher, D3821) was added to the confocal dishes and incubated with the cells for 1 h. Following incubation, the cell nuclei were stained with Hoechst for 8 min. Uptake of FA was monitored using flow cytometry (CytoFLEX, Beckman Coulter, USA) and DeltaVision OMX (GE, USA).

### Energy Metabolism Analysis

Cell energy metabolism analysis was conducted using a Seahorse cell energy metabolism analyzer (Agilent, USA). In brief, 8000 cells were seeded per well in a 96‐well XF96 plate (Agilent, 103794‐100) and cultured overnight. Subsequently, the cells were treated with various concentrations of peptides for 24 h. To prepare for metabolic stress injections, cartridge plates (Agilent, w15023) were hydrated with H_2_O for at least 12 h at 37 °C before the assay. 1 h before detection, the medium in each well was replaced with 180 µL XF calibrant solution (Agilent, 100840‐000), Afterward, the cell plate was replaced with 180 µL XF DMEM (Agilent, 103575‐100) with glucose (1 × 10^−2^ M) (Agilent, 103577‐100), pyruvate (1 × 10^−3^ M) (Agilent, 103578‐100) and glutamine (2 × 10^−3^ M) (Agilent, 103579‐100) and the mixture was incubated in a CO_2_‐free incubator at 37 °C for 1 h. Then, 20 µL basic medium, 22 µL 1.5 × 10^−4^ M oligomycin, 25 µL 1.5 × 10^−4^ M FCCP, and 27 µL 5 × 10^−5^ M ROT/AA (Agilent, 10315‐100) were added to wells A, B, C, and D, respectively, of the cartridge plates. The assay was carried out according to the manufacturer's instructions, and the OCR was reported in picomoles per min. The results were normalized to the cell number determined by the CCK‐8 assay.

### Scratch Assay

MCF7 and MDA‐MB‐231 cells were seeded in 6‐well plates and cultured overnight. Once the cell density reached 90%, a line was scratched along the center of the 6‐well plate and images were taken randomly in the scratch domain under a microscope. The cells in each well were then treated with peptides of varying concentrations for 12 h, after which images were taken again in the scratch domain. The formula used to calculate the scratch healing rate was as follows: Scratch healing rate (%) = [(Scratch area at 0 h – Scratch area at 12 h) – Scratch area at 0 h] × 100%.

### Cell Migration Analysis

600 µL DMEM complete medium was added to the lower chamber of the Transwell. Next, 2×10^4^ cells in 100 µL DMEM medium, containing 1, 2, 5, 10, or 2 × 10^−5^ M peptides, were added to the upper chamber of the Transwell. The cells were incubated for 12 h at 37 °C and 5% CO_2_. After the incubation period, the medium was discarded, and the chamber was washed with PBS. The cells were then fixed with 4% paraformaldehyde for 20 min. The upper chamber was subsequently soaked in crystal violet for 20 min, washed with PBS, and observed under a microscope for analysis.

### Animal Experiment

To establish the bone metastasis tumor model, 1×10^5^ cells were injected into the left ventricle of each mouse (7–8 weeks old), and surgery was monitored using a cardiac ultrasound instrument. The mice were randomly divided into three groups and treated with PBS (control group, n = 6), 5 mg kg^−1^ ST‐CY14 (n = 6), and 10 mg kg^−1^ ST‐CY14 (n = 6) Tumor cell metastasis was monitored using an in vivo imaging instrument (IVIS Spectrum, PerkinElmer, USA). by intraperitoneal injection of 20 mg kg^−1^ D‐luciferin. The mice were treated with ST‐CY14 via intravenous injection using a 100 µL PBS solution every 2 days starting on the designated day after tumor cell injection. Bone tissue with MDA‐BoM‐1833 cell metastasis was obtained and stained for TRAP. The mice sera were collected and analyzed for BSP and β‐CTx levels using ELISA (Lengton, BPE10823, BPE10438). For the orthotopic xenograft tumor model, 3×10^6^ cells were injected into the fourth pair of breast pads on the left side of the mouse (4–5 weeks old). The mice were treated with ST‐CY14 every two days once the tumor volume reached 30–50 mm^3^, with the same groups as in the bone metastasis tumor model. The tumors were collected and stained for H&E, Ki‐67, and TUNEL assays, which were conducted by Wuhan Servicebio Technology Co., Ltd. Major organs (heart, liver, spleen, lung, and kidney) were also collected for H&E analysis by Wuhan Servicebio Technology Co., Ltd. The Transcriptome and LC/MS targeted lipid metabolome were sequenced by OE Biotech, Inc., Shanghai, China. To evaluate the efficacy of Csn‐B in inhibiting breast cancer bone metastasis, an animal model was established as described above. The mice were randomly divided into three groups and treated with PBS (control group, n = 6), 10 mg kg^−1^ ST‐CY14 (n = 6) in 100 µL PBS solution, and 5 mg kg^−1^ ST‐CY14 (n = 6) in a 100 µL mixed solution (5% DMSO, 20% PEG 400, 5% Tween 80, and 70% normal saline) via intravenous injection every 2 days starting on the designated day after tumor cell injection.

### Statistical Analysis

All the statistical analyses were performed with GraphPad Prism 8.0 software. All the data were shown as the mean ± standard deviation (S.D.). Statistical analysis was performed with Student's t‐test or ANOVA with Tukey's multiple comparison tests.

## Conflict of Interest

The authors declare no conflict of interest.

## Supporting information

Supporting Information

Supporting Information

Supporting Information

## Data Availability

The data that support the findings of this study are available in the supplementary material of this article.
