# Supplementary material for: In Silico Discovery of Stapled Peptide Inhibitor Targeting the Nur77‐PPARγ Interaction and Its Anti‐Breast‐Cancer Efficacy
Source: Adv Sci (Weinh). 2024 Apr 29;11(26):2308435. doi: 10.1002/advs.202308435 (PMC11234460; doi:10.1002/advs.202308435)
Supplement: Supplementary file 1 — Supporting Information [file ADVS-11-2308435-s003.docx]

Supporting Information

*In Silico* discovery of stapled peptide inhibitor targeting the Nur77-PPARγ interaction and its anti-breast-cancer efficacy

Huiting Bian, Xiaohui Liang, Dong Lu, Jiayi Lin, Xinchen Lu, Jinmei Jin, Lijun Zhang, Ye Wu,* Hongzhuan Chen,* Weidong Zhang,* and Xin Luan*

H.-T. Bian, X.-H. Liang, D. Lu, J.-Y. Lin, X.-C. Lu, J.-M. Jin, L.-J. Zhang, Y. Wu, H.-Z. Chen, W.-D. Zhang, X. Luan

Shanghai Frontiers Science Center of TCM Chemical Biology, Institute of Interdisciplinary Integrative Medicine Research and Shuguang Hospital, Shanghai University of Traditional Chinese Medicine, Shanghai 201203, China
E-mail: [wuye@shutcm.edu.cn](mailto:wuye@shutcm.edu.cn); [wdzhangy@hotmail.com](mailto:wdzhangy@hotmail.com); hongzhuan_chen@hotmail.com; [luanxin@shutcm.edu.cn](mailto:luanxin@shutcm.edu.cn)

H.-T. Bian, X.-C. Lu, W.-D. Zhang

School of Pharmacy, Fudan University, Shanghai 201203, China

W.-D. Zhang

School of Pharmacy, Second Military Medical University, Shanghai 200433, China

W.-D. Zhang

Institute of Medicinal Plant Development, Chinese Academy of Medical Science &, Peking Union Medical College, Beijing 100193, China

**Table of Contents**

[**Table S1.** The sequence of siRNA. 3](#_Toc157276038)

[**Table S2.** The sequence, molecular formula, molecular weight, and observed MS of peptides. 4](#_Toc157276039)

[**Figure S1.** The correlation between the overall survival of breast cancer patients and Nur77/PPARγ expression. 5](#_Toc157276040)

[**Figure S2.** *In silico* systematic approach to predict the structure of the Nur77-PPARγ heterodimer. 5](#_Toc157276041)

[**Figure S3.** Identification of inhibitors targeting the Nur77-PPARγ interaction in rank2 and rank3. 6](#_Toc157276042)

[**Figure S4.** The binding affinity of CY14 derivatives with Nur77 7](#_Toc157276043)

[**Figure S5.** The blocking effect of ST-CY14 on Nur77-PPARγ interaction 7](#_Toc157276044)

[**Figure S6.** The cell viability of breast tumor cells after treatment with ST-CY14 or Csn-B. 8](#_Toc157276045)

[**Figure S7.** The cell viability of Nur77-knockdown breast tumor cells and normal breast cells after treatment with ST-CY14. 8](#_Toc157276046)

[**Figure S8.** Comparison of the solubility between ST-CY14 and Csn-B. 8](#_Toc157276047)

[**Figure S9.** Representative images of H&E staining of major organs in MDA-MB-231-GFP xenograft model. 9](#_Toc157276048)

[**Figure S10.** Representative images of Ki-67, H&E, and TUNEL staining of MDA-MB-231-GFP tumors. . 9](#_Toc157276049)

[**Figure S11.** Representative images of H&E staining of major organs in bone metastatic breast cancer model. 10](#_Toc157276050)

[**Figure S12.** Comparison of the efficacy of ST-CY14 and Csn-B in inhibiting breast cancer bone metastasis. 10](#_Toc157276051)

[**Figure S13.** HPLC of compound SV16. 11](#_Toc157276052)

[**Figure S14.** MS of compound SV16. 11](#_Toc157276053)

[**Figure S15.** HPLC of compound CL14. 11](#_Toc157276054)

[**Figure S16.** MS of compound CL14. 11](#_Toc157276055)

[**Figure S17.** HPLC of compound CY14. 12](#_Toc157276056)

[**Figure S18.** MS of compound CY14. 12](#_Toc157276057)

[**Figure S19.** HPLC of compound FITC-CY14. 12](#_Toc157276058)

[**Figure S20.** MS of compound FITC-CY14. 12](#_Toc157276059)

[**Figure S21.** HPLC of compound CF14. 13](#_Toc157276060)

[**Figure S22.** MS of compound CF14. 13](#_Toc157276061)

[**Figure S23.** HPLC of compound CH14. 13](#_Toc157276062)

[**Figure S24.** MS of compound CH14. 13](#_Toc157276063)

[**Figure S25.** HPLC of compound CW14. 14](#_Toc157276064)

[**Figure S26.** MS of compound CW14. 14](#_Toc157276065)

[**Figure S27.** HPLC of compound TAT. 14](#_Toc157276066)

[**Figure S28.** MS of compound TAT. 14](#_Toc157276067)

[**Figure S29.** HPLC of compound T-CY14. 15](#_Toc157276068)

[**Figure S30.** MS of compound T-CY14. 15](#_Toc157276069)

[**Figure S31.** HPLC of compound FITC-T-CY14. 15](#_Toc157276070)

[**Figure S32.** MS of compound FITC-T-CY14. 15](#_Toc157276071)

[**Figure S33.** HPLC of compound S-CY14. 16](#_Toc157276072)

[**Figure S34.** MS of compound S-CY14. 16](#_Toc157276073)

[**Figure S35.** HPLC of compound ST-CY14. 16](#_Toc157276074)

[**Figure S36.** MS of compound ST-CY14. 16](#_Toc157276075)

**Table S1.** The sequence of siRNA.

| **Name** | **sequence** |
| --- | --- |
| si-Nur77 | sense (5'-3') UGGUGAAGGAAGUUGUCCGAAtt |
|  | anti-sense (5'-3') UUCGGACAACUUCCUUCACCAtt |
| siPPARγ1 | sense (5'-3') ACUCCACAUUACGAAGACATT |
|  | anti-sense (5'-3') UGUCUUCGUAAUGUGGAGUTT |
| siPPARγ2 | sense (5'-3') CUGGCCUCCUUGAUGAAUATT |
|  | anti-sense (5'-3') UAUUCAUCAAGGAGGCCAGTT |

**Table S2.** The sequence, molecular formula, molecular weight, and observed MS of peptides.

| **Peptide** | **Sequence** | **Molecular formula** | **Molecular Weight (g/mol)** | **Observed MS (m/q)** |
| --- | --- | --- | --- | --- |
| SV16 | H-SRNKCQYCRFQKCLAV-NH_2_ | C_82_H_136_N_28_O_21_S_3_ | 1946.35 | [M+2H]^2+^=973.90;  [M+3H]^3+^=649.70;  [M+4H]^4+^=487.60; |
| CL14 | H-CEGCKGFFRRTIRL-NH_2_ | C_73_H_121_N_25_O_17_S_2_ | 1685.05 | [M+2H]^2+^=843.30;  [M+3H]^3+^=562.60;  [M+4H]^4+^=422.20; |
| CY14 | H-CEGCKGFFRRTIRY-NH_2_ | C_76_H_119_N_25_O_18_S_2_ | 1735.07 | [M+2H]^2+^=868.30;  [M+3H]^3+^=579.40;  [M+4H]^4+^=434.80; |
| FITC-CY14 | FITC-Ahx-CEGCKGFFRRTIRY-NH_2_ | C_103_H_141_N_27_O_24_S_3_ | 2237.61 | [M+3H]^3+^=746.80;  [M+4H]^4+^=560.40;  [M+5H]^5+^=448.50; |
| CF14 | H-CEGCKGFFRRTIRF-NH_2_ | C_76_H_119_N_25_O_17_S_2_ | 1719.09 | [M+2H]^2+^=860.30;  [M+3H]^3+^=573.90;  [M+4H]^4+^=430.90; |
| CH14 | H-CEGCKGFFRRTIRH-NH_2_ | C_73_H_117_N_27_O_17_S_2_ | 1709.03 | [M+2H]^2+^=855.20;  [M+3H]^3+^=570.60;  [M+4H]^4+^=428.20; |
| CW14 | H-CEGCKGFFRRTIRW-NH_2_ | C_78_H_120_N_26_O_17_S_2_ | 1758.10 | [M+2H]^2+^=878.70;  [M+3H]^3+^=586.20;  [M+4H]^4+^=439.90; |
| TAT | H-YGRKKRRQRRR-NH_2_ | C_64_H_119_N_33_O_13_ | 1558.87 | [M+2H]^2+^=780.30;  [M+3H]^3+^=520.60;  [M+4H]^4+^=390.80;  [M+5H]^5+^=312.80; |
| T-CY14 | H-CEGCKGFFRRTIRYYGRKKRRQRRR-NH_2_ | C_140_H_235_N_57_O_31_S_2_ | 3276.91 | [M+4H]^4+^=820.50;  [M+5H]^5+^=656.50;  [M+6H]^6+^=547.30;  [M+7H]^7+^=469.20;  [M+8H]^8+^=410.70; |
| FITC-T-CY14 | FITC-Ahx-CEGCKGFFRRTIRYYGRKKRRQRRR-NH_2_ | C_167_H_257_N_59_O_37_S_3_ | 3779.45 | [M+4H]^4+^=946.00;  [M+5H]^5+^=757.20;  [M+6H]^6+^=631.10;  [M+7H]^7+^=541.30;  [M+8H]^8+^=473.80; |
| S-CY14 | H-CEGCKGFXRRTXRY-NH_2_ | C_75_H_121_N_25_O_18_S_2_ | 1725.07 | [M+2H]^2+^=863.60;  [M+3H]^3+^=576.20;  [M+4H]^4+^=432.50; |
| ST-CY14 | H-CEGCKGFXRRTXRYYGRKKRRQRRR-NH_2_ | C_139_H_237_N_57_O_31_S_2_ | 3266.91 | [M+3H]^3+^=1089.70;  [M+4H]^4+^=817.70;  [M+5H]^5+^=654.30;  [M+6H]^6+^=545.50;  [M+7H]^7+^=467.70; |

Note: H and NH_2_ in the peptide sequence represent the N-terminal amino group and C-terminal primary amide, respectively.


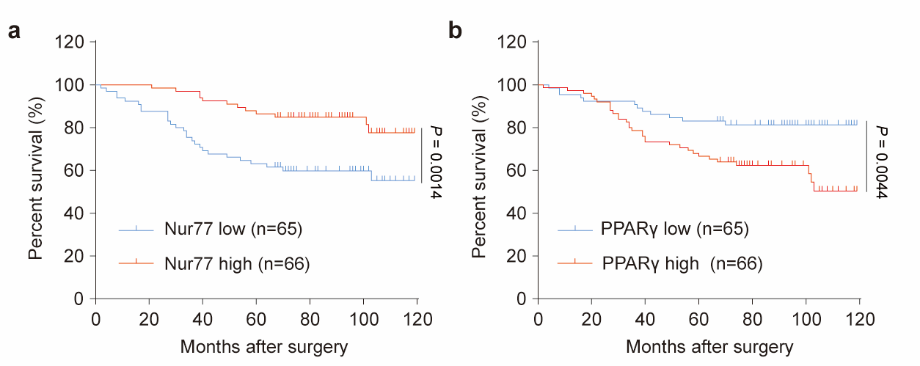


**Figure S1.** Kaplan–Meier survival curve showing the correlation between the overall survival of breast cancer patients and a) Nur77, b) PPARγ expression. The *P* values were calculated by the log-rank (Mantel-Cox) test.


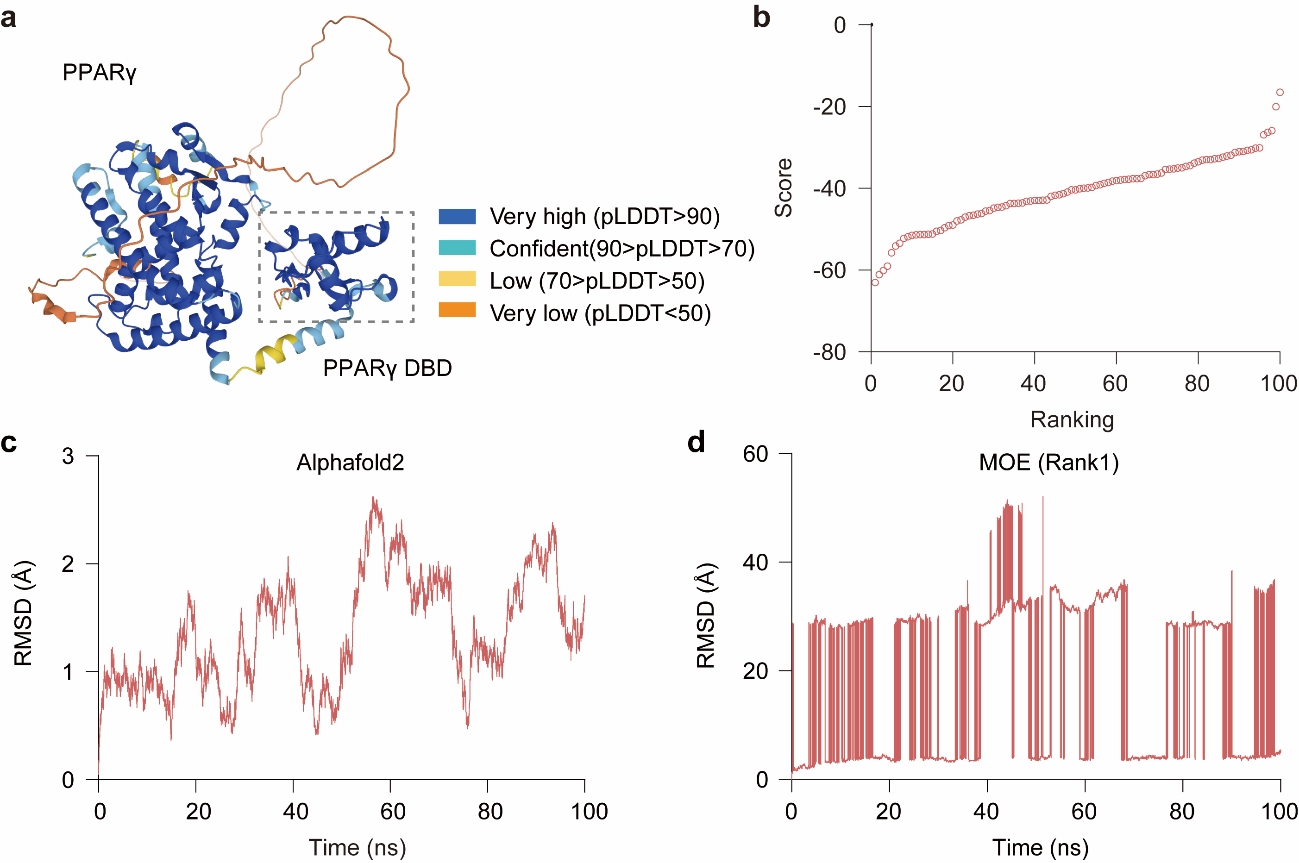


**Figure S2.** *In silico* systematic approach to predict the structure of the Nur77-PPARγ heterodimer. a) The structure of the PPARγ DBD predicted by AlphaFold2. b) Docking score ranking of the interaction between Nur77 and PPARγ. c) Cα RMSD of the structure predicted by AlphaFold2. d, Cα RMSD of the rank 1 structure predicted by MOE.

**
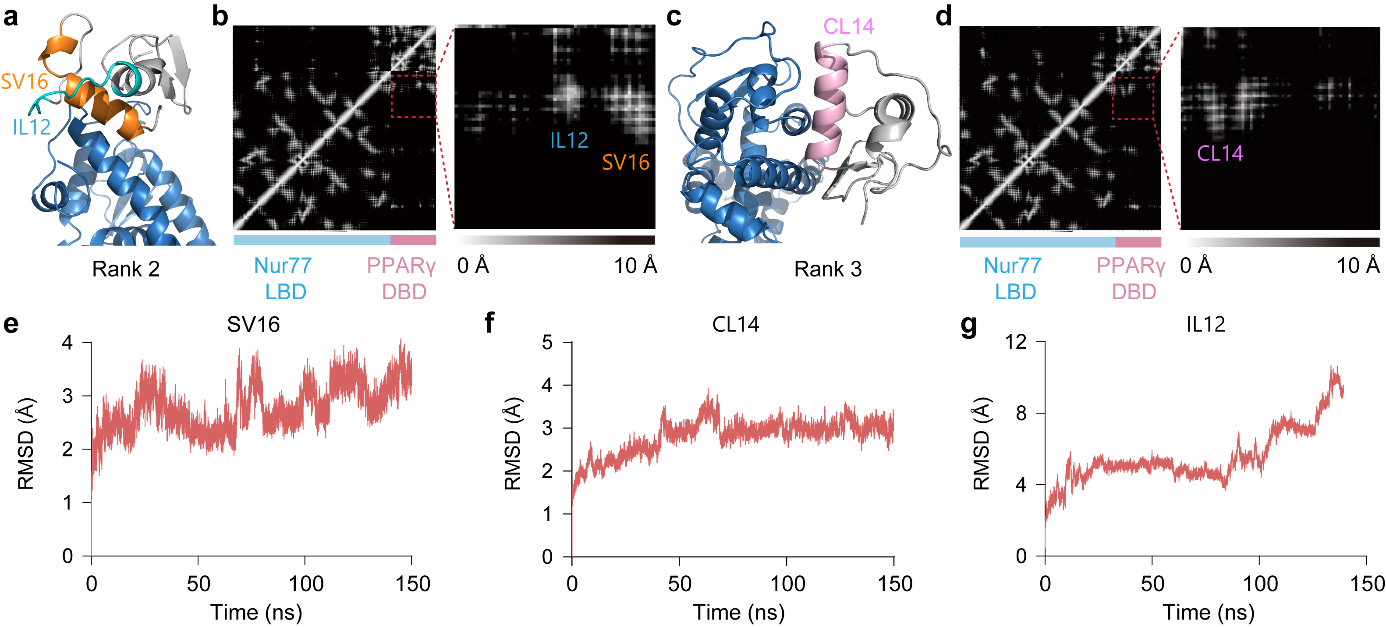
**

**Figure S3.** Identification of inhibitors targeting the Nur77-PPARγ interaction. a) Cartoon representation of the peptides SV16 (colored orange) and IL12 (colored blue) bound to the binding surface of Nur77 and PPARγ in rank 2. b) Contact map illustrating the interactions between Nur77 and PPARγ in rank 2. c) The peptide CL14 (colored pink) bound to the binding surface of Nur77 and PPARγ in rank 3 is depicted in cartoon representation. d) The contact map illustrating the interactions between Nur77 and PPARγ in rank 3. e-g) The Cα RMSD values depicting the structural deviation of e) SV16, f) CL14, and g) IL12.

**
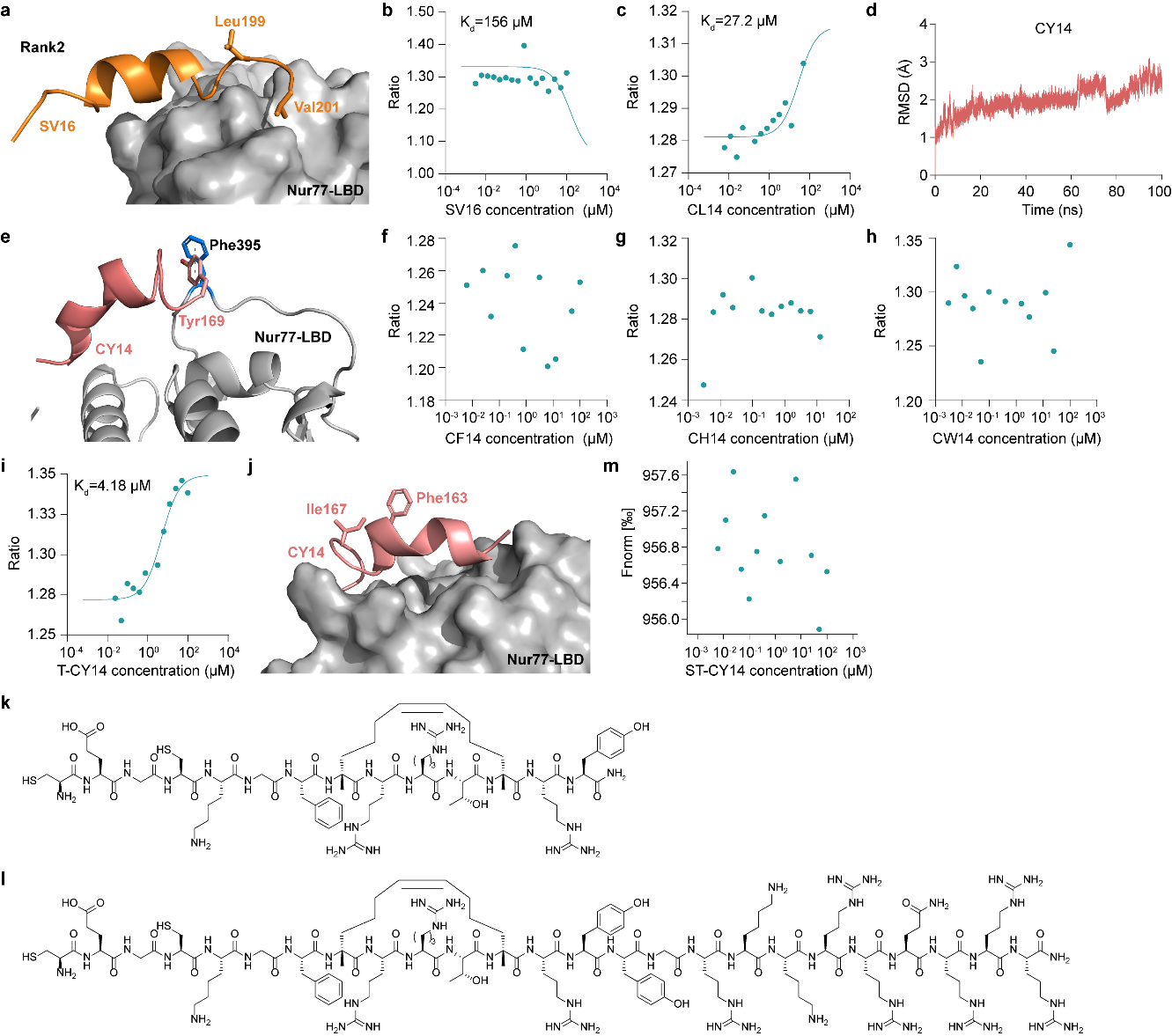
**

**Figure S4.** The binding affinity of CY14 derivatives with Nur77. a) The interaction between SV16 Val201 and the Nur77 LBD. The binding affinity of b) SV16, c) CL14, d) CF14, e) CH14, f) CW14 with Nur77-LBD. g) The Cα RMSD of the CY14-Nur77 conformation. h) The interactions between CY14 Tyr169 and the Nur77 LBD. i) The Phe163 and Ile167 of CY14 are exposed to the solvent surface when CY14 binds to the Nur77 LBD. j) The binding affinity of T-CY14 with Nur77-LBD. k) The chemical structure of S-CY14. l) The chemical structure of ST-CY14. m) The affinity of ST-CY14 with mutated Nur77 is depicted using MST.


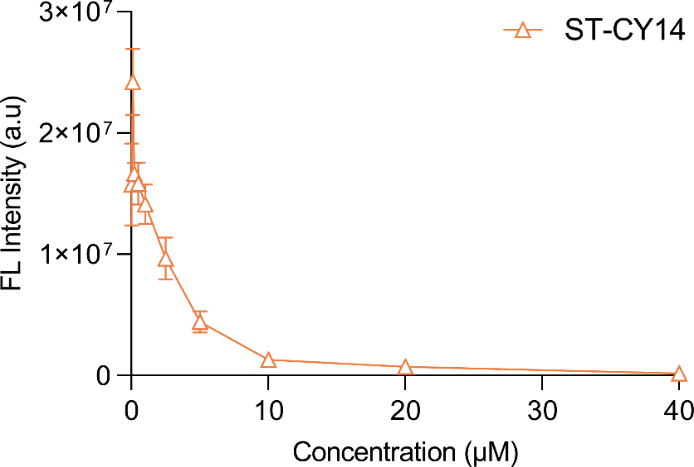


**Figure S5.** The blocking effect of ST-CY14 on the Nur77-PPARγ interaction was detected via NanoBit.


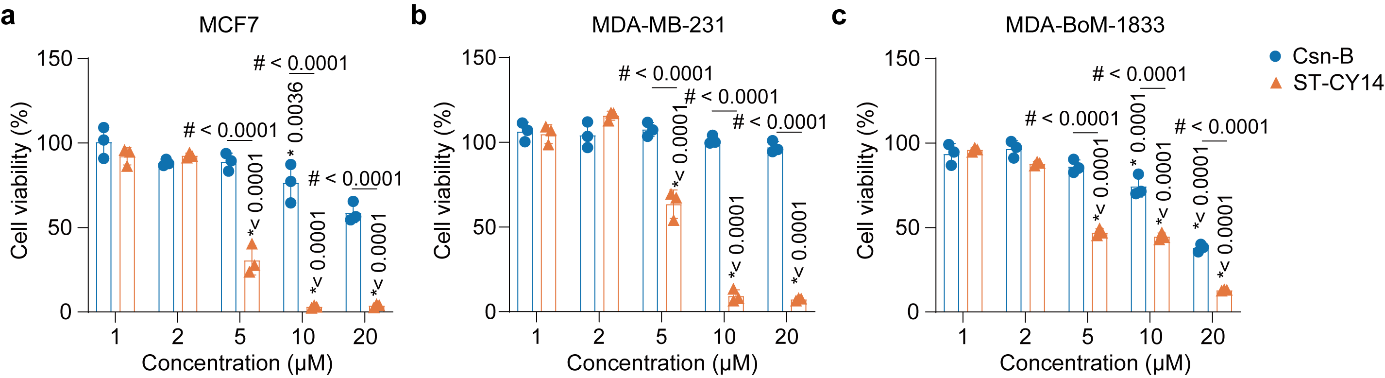


**Figure S6.** The cell viability of a) MCF7, b) MDA-MB-231, and c) MDA-BoM-1833 cells after treatment with ST-CY14 or Csn-B for 24 h.


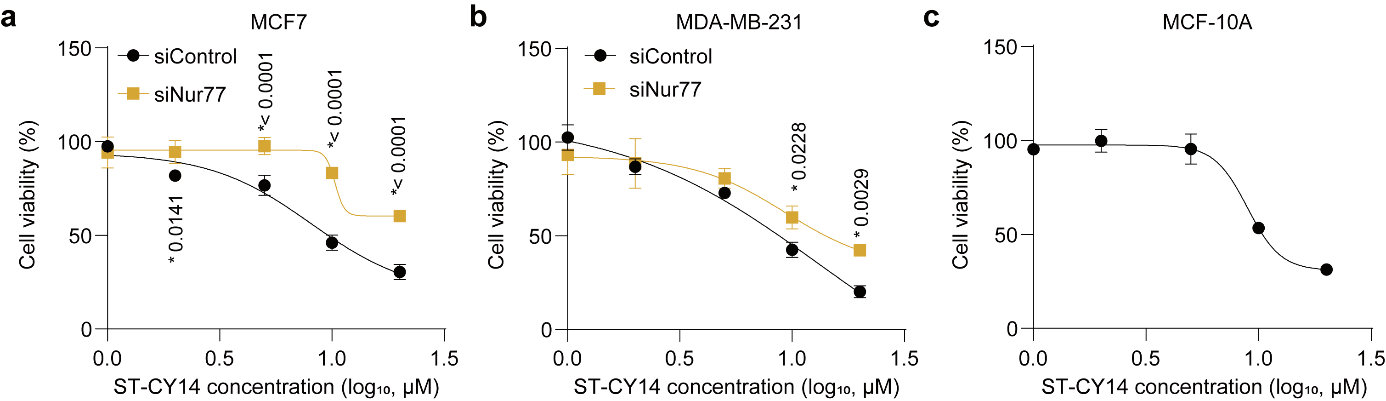


**Figure S7.** The cell viability of a) Nur77-knockdown MCF7, b) Nur77-knockdown MDA-MB-231, and c) normal breast cells (MCF-10A) after treatment with ST-CY14 for 24 h.


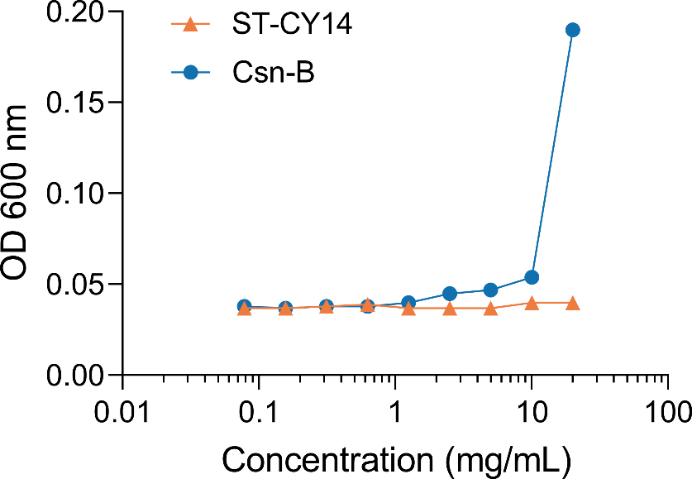


**Figure S8.** Comparison of the solubility between ST-CY14 and Csn-B.


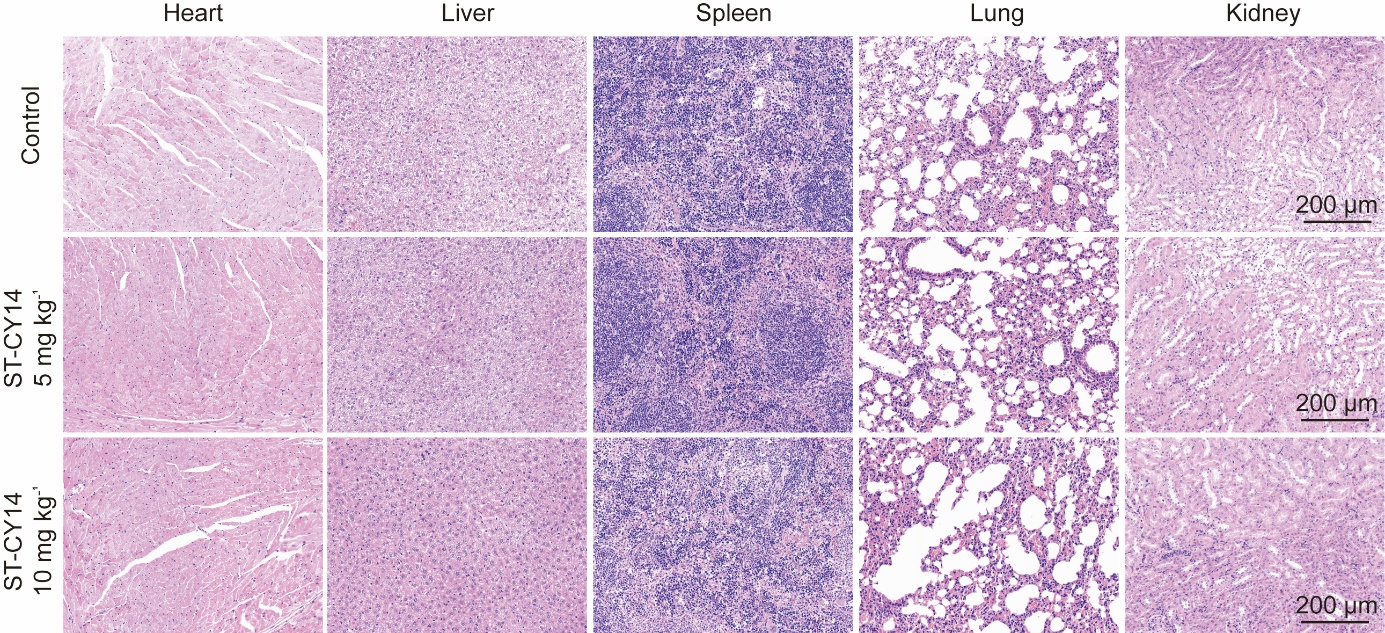


**Figure S9.** Representative images of H&E staining of heart, liver, spleen, lung, and kidney tissues collected from different groups in the MDA-MB-231-GFP xenograft model at the end of observation (n=3).


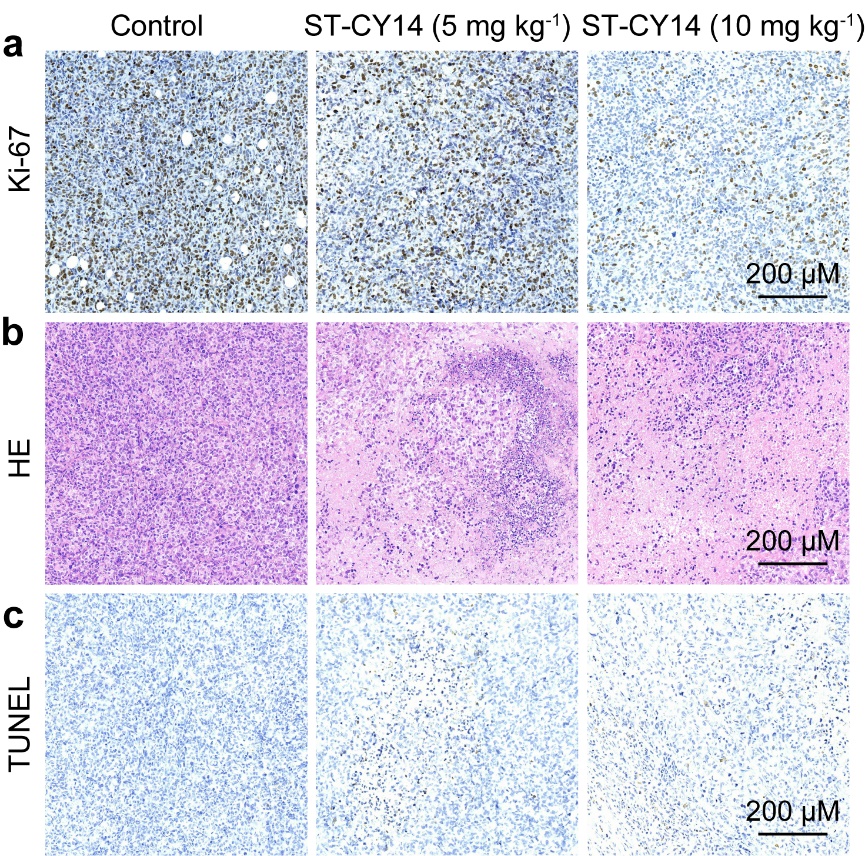


**Figure S10.** Representative images of a) Ki-67, b) H&E, and c) TUNEL staining of MDA-MB-231-GFP tumor samples collected from different groups at the end of observation (n=3).


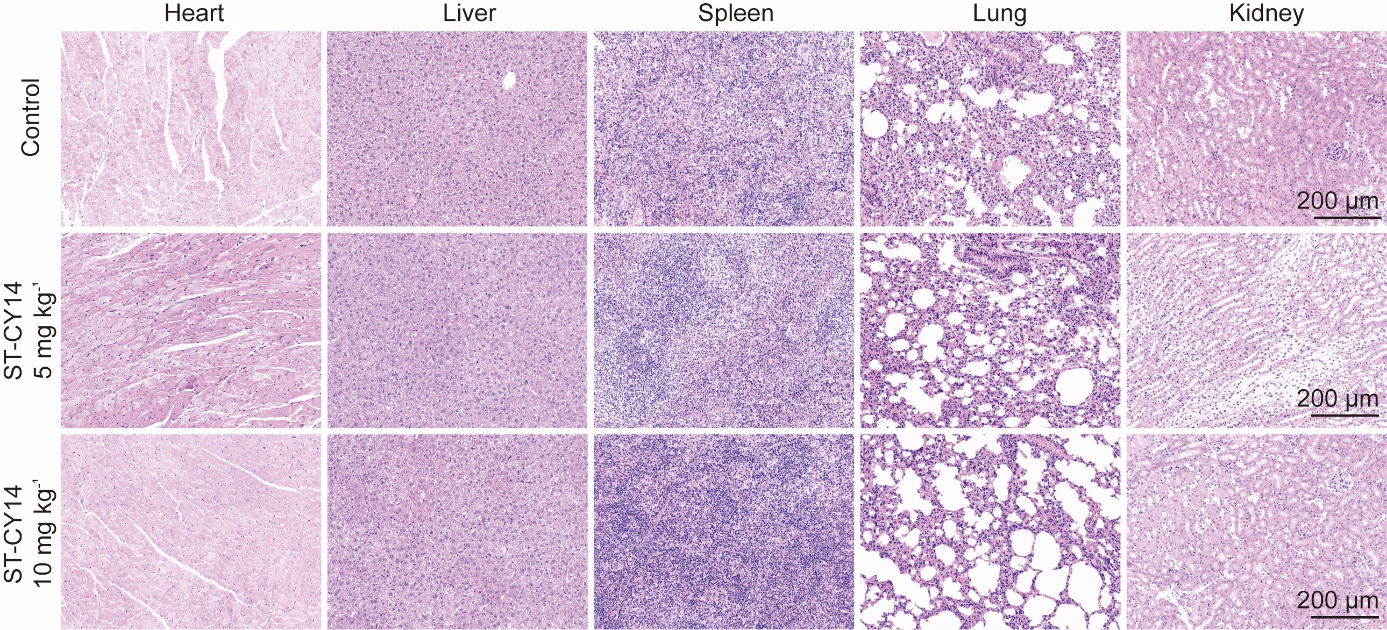


**Figure S11.** Representative images of H&E staining of heart, liver, spleen, lung, and kidney tissues from different groups in the bone metastatic breast cancer model at the end of observation (n=3).


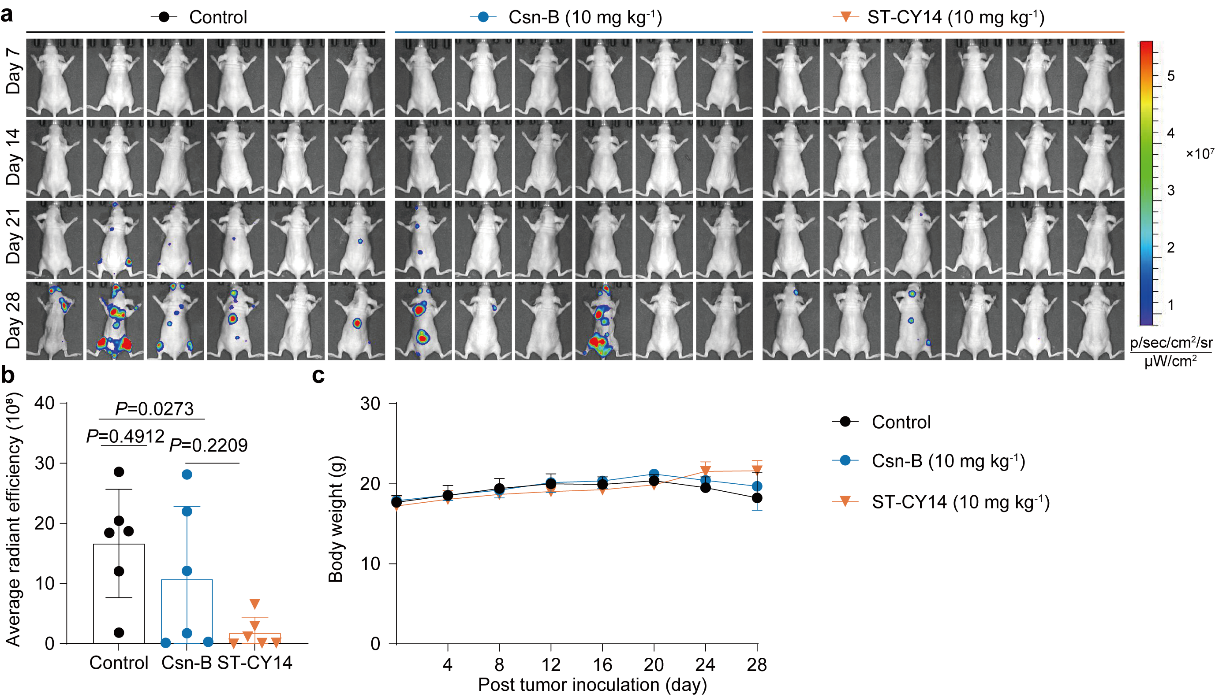


**Figure S12.** Comparison of the efficacy of ST-CY14 and Csn-B in inhibiting breast cancer bone metastasis. a) In vivo imaging of mice every week. b) Bone metastasis tumor accumulated optical density measurement on day 28 (n=6), the quantified data from different experiments were presented as the mean ± SD. The P values were calculated by one-way ANOVA. c) Time-course of body weight (n=6).


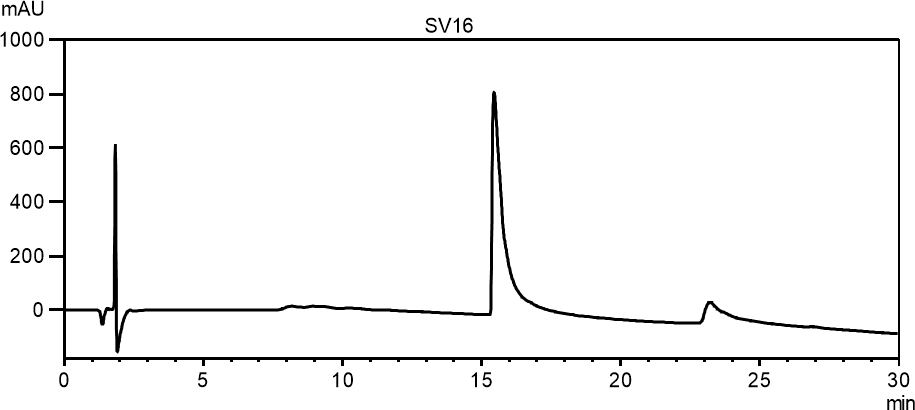


**Figure S13.** HPLC of compound SV16.


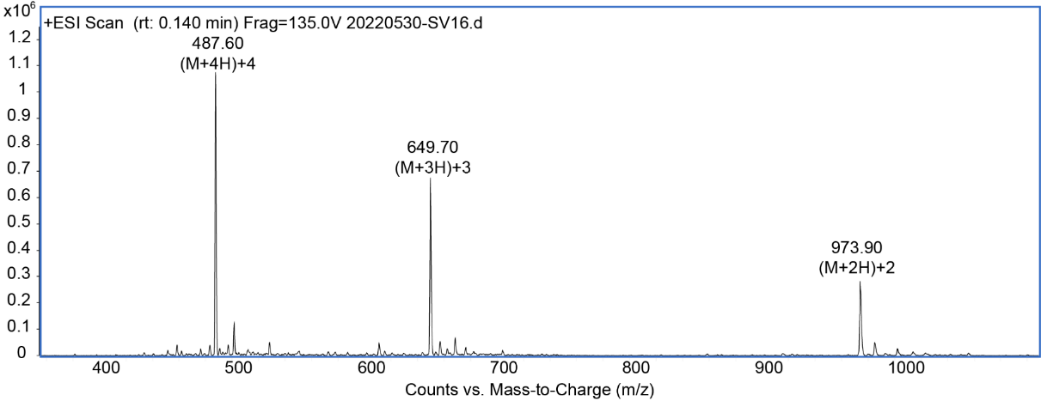


**Figure S14.** MS of compound SV16.


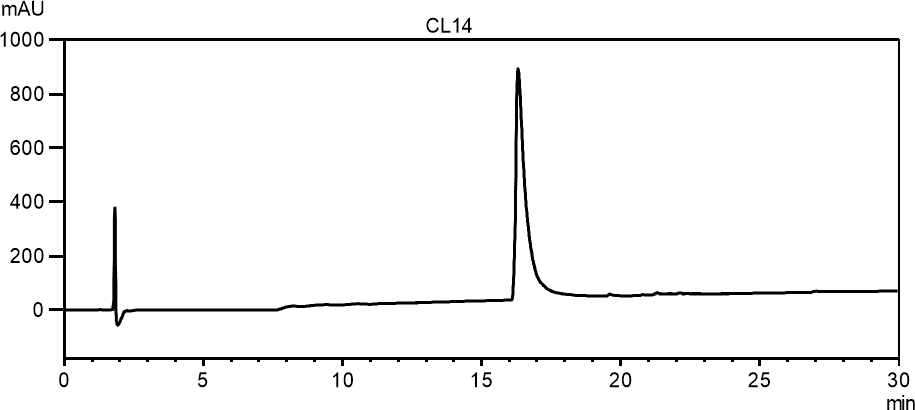


**Figure S15.** HPLC of compound CL14.


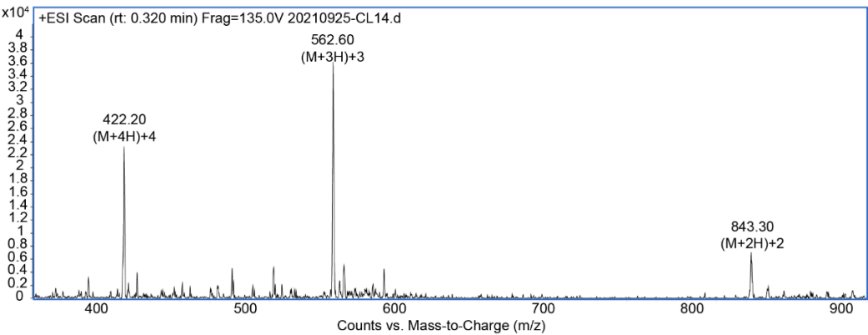


**Figure S16.** MS of compound CL14.


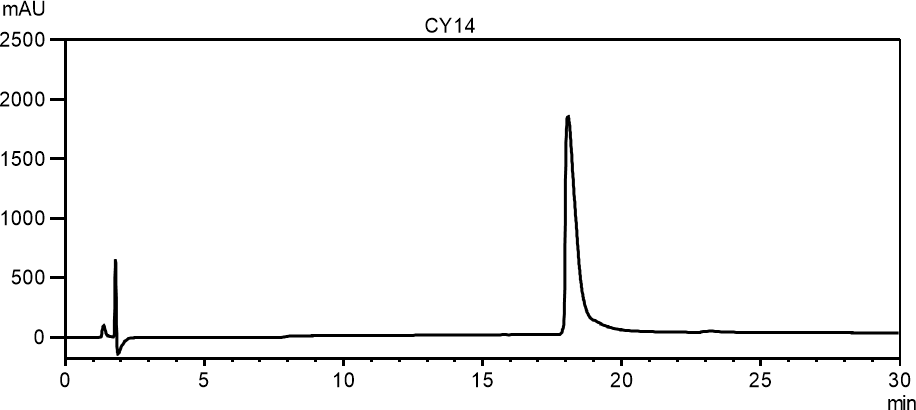


**Figure S17.** HPLC of compound CY14.


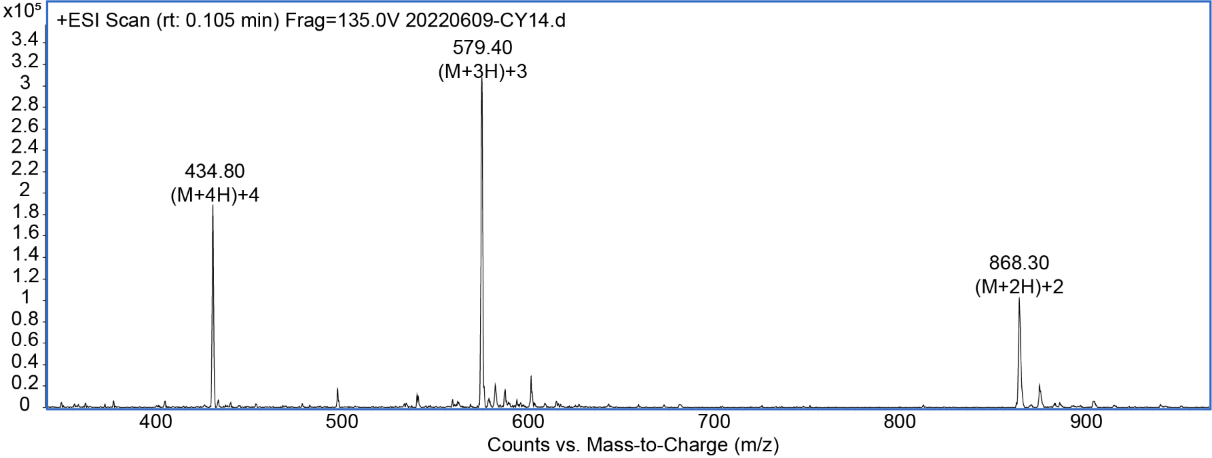


**Figure S18.** MS of compound CY14.


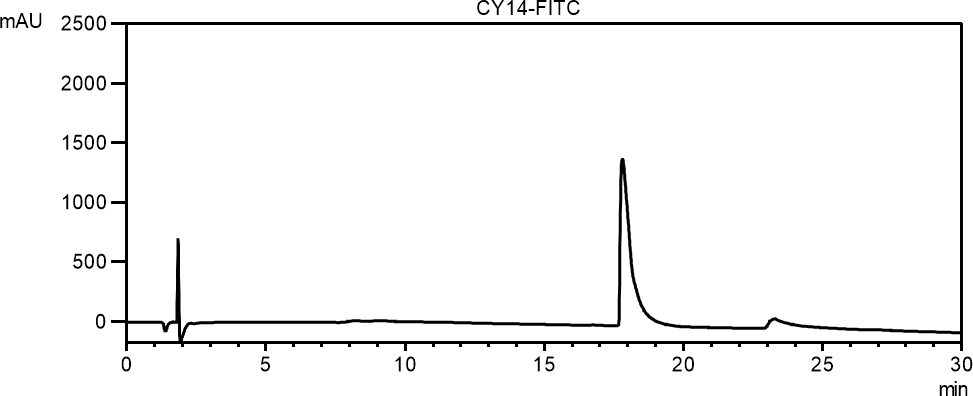


**Figure S19.** HPLC of compound FITC-CY14.


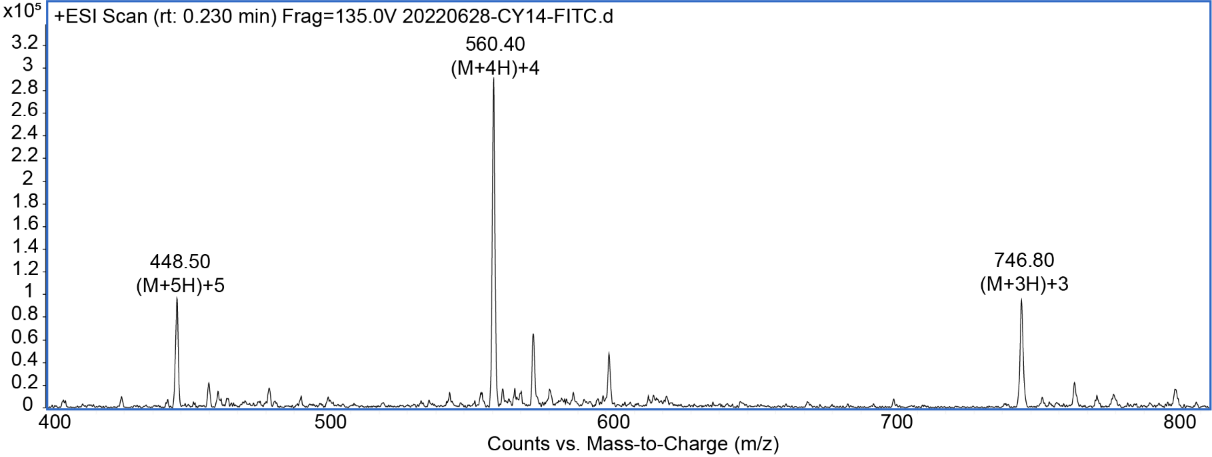


**Figure S20.** MS of compound FITC-CY14.


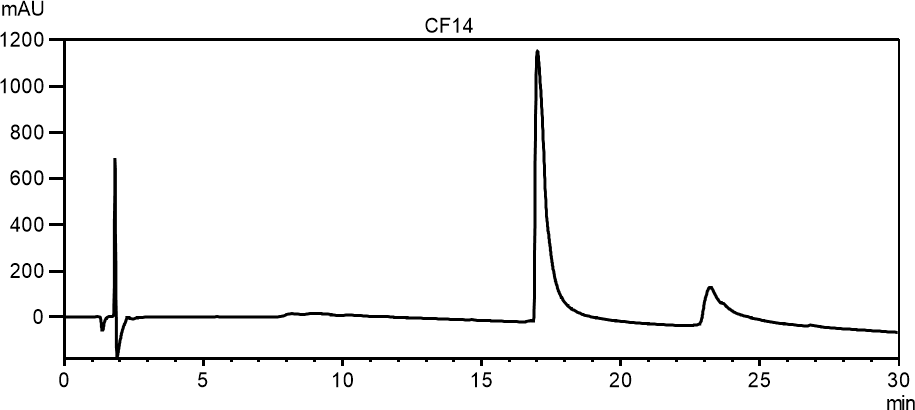


**Figure S21.** HPLC of compound CF14.


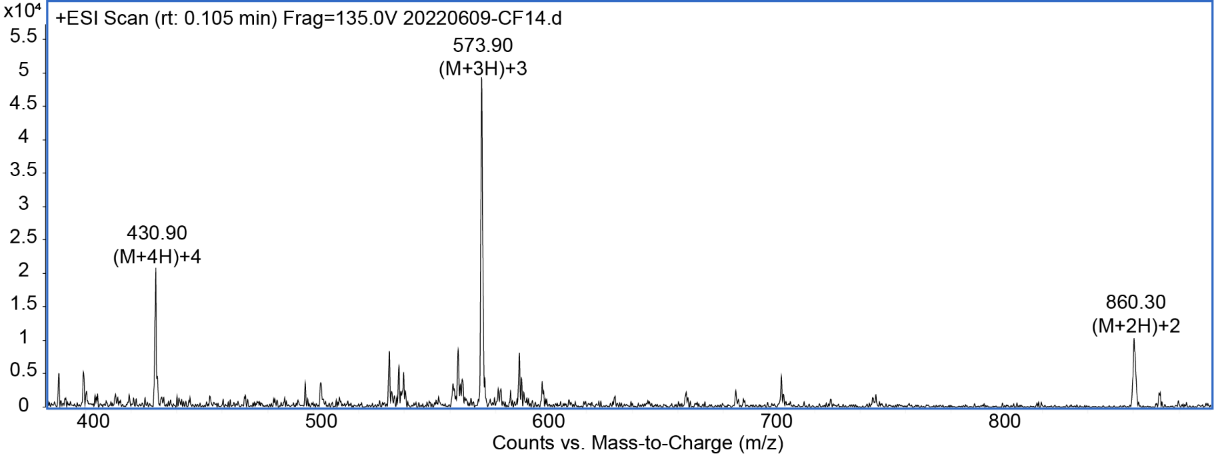


**Figure S22.** MS of compound CF14.


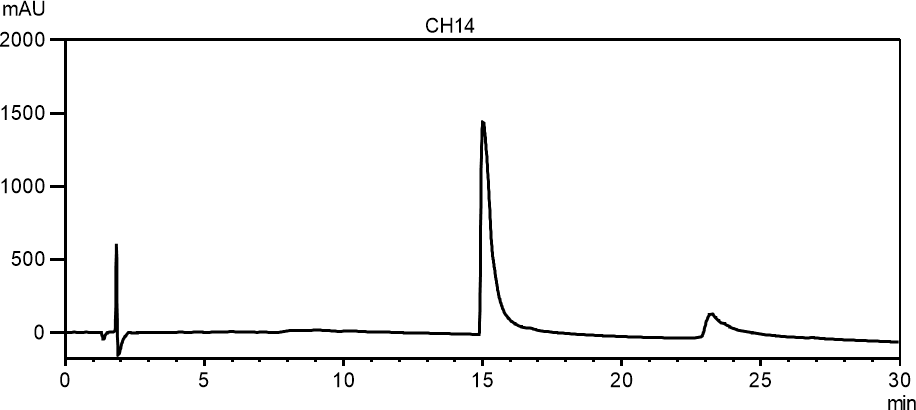


**Figure S23.** HPLC of compound CH14.


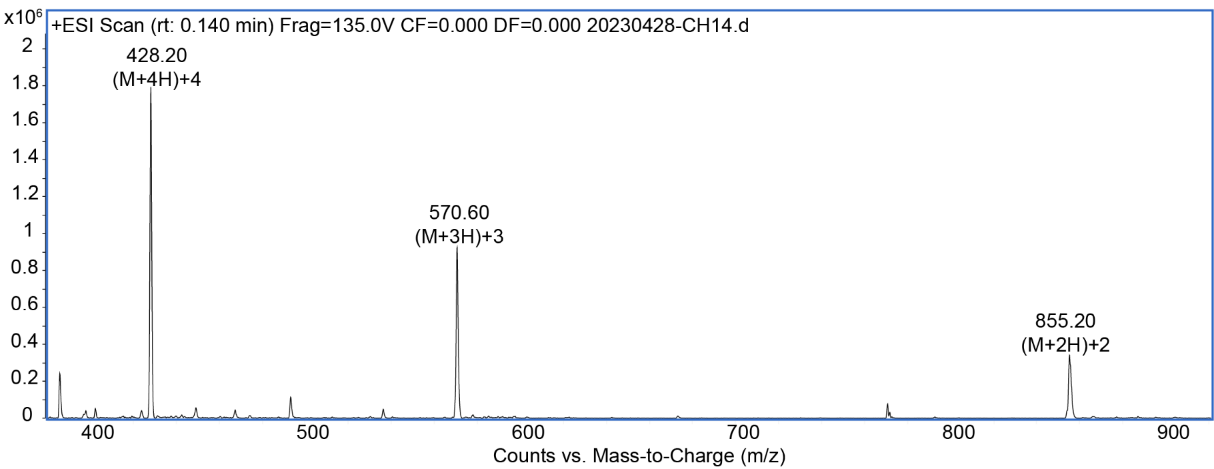


**Figure S24.** MS of compound CH14.


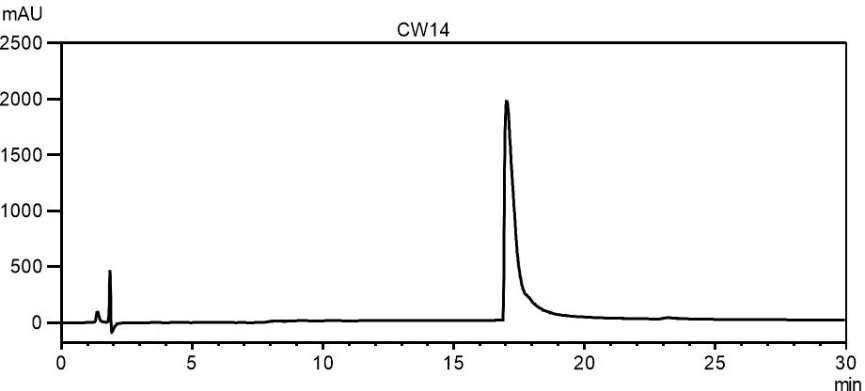


**Figure S25.** HPLC of compound CW14.


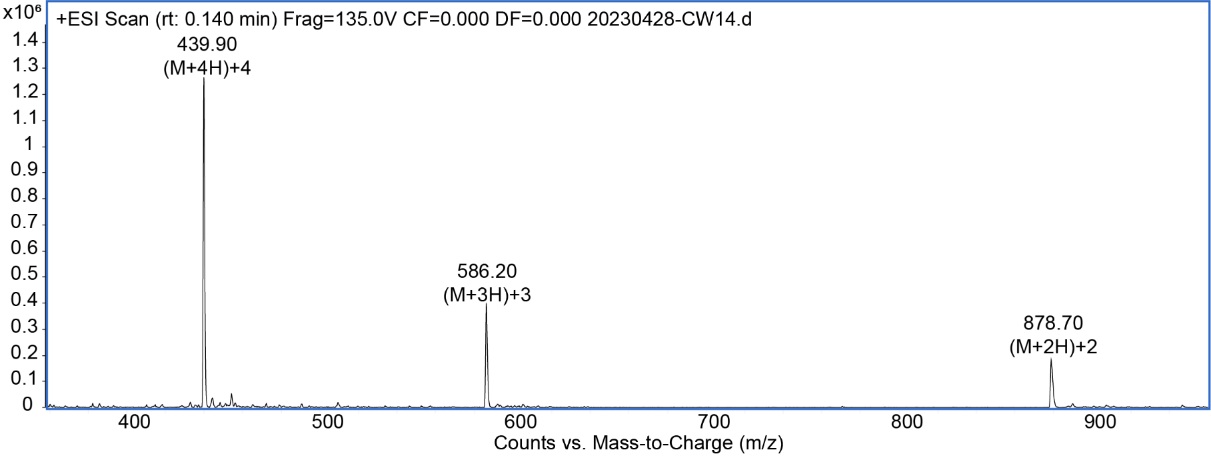


**Figure S26.** MS of compound CW14.


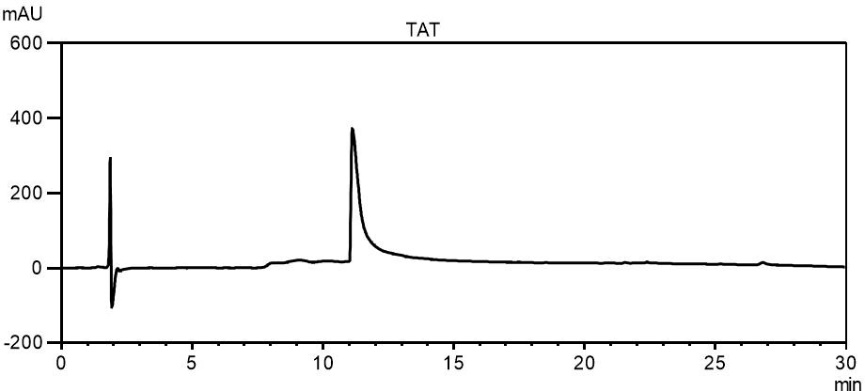


**Figure S27.** HPLC of compound TAT.


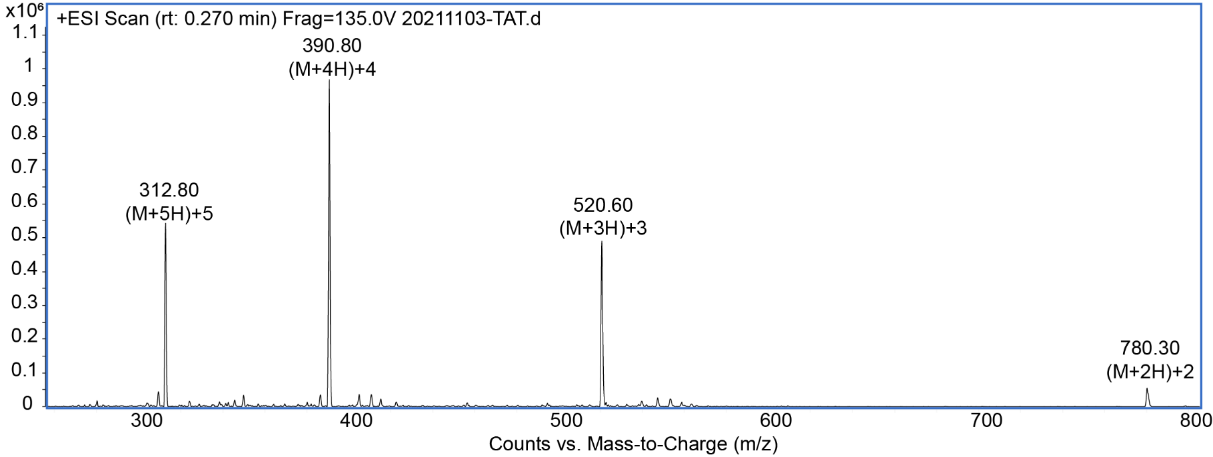


**Figure S28.** MS of compound TAT.


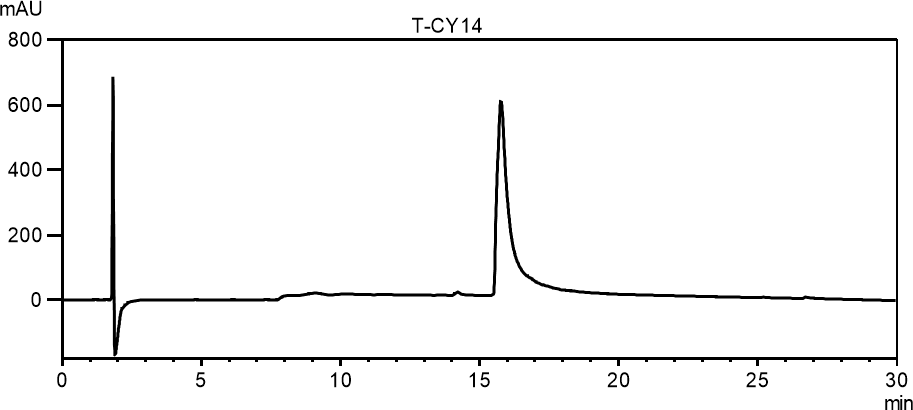


**Figure S29.** HPLC of compound T-CY14.


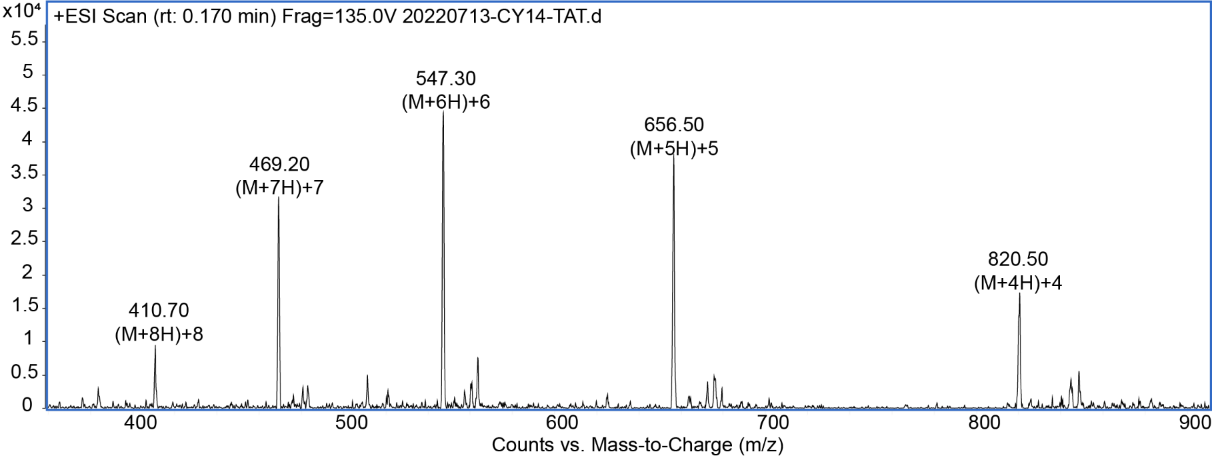


**Figure S30.** MS of compound T-CY14.


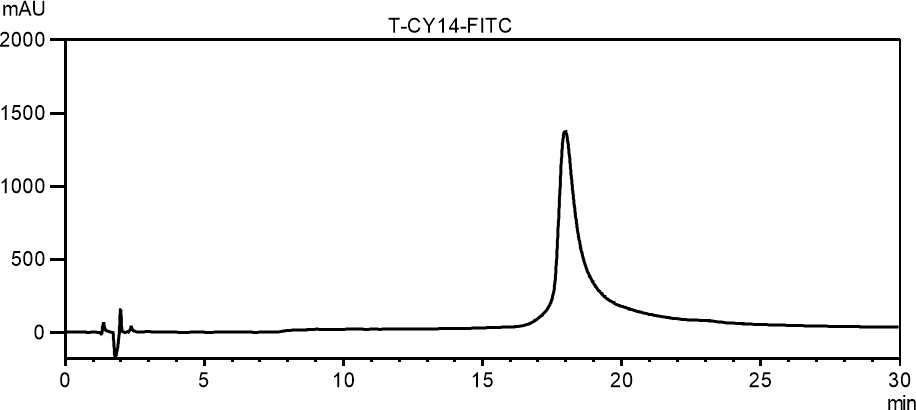


**Figure S31.** HPLC of compound FITC-T-CY14.


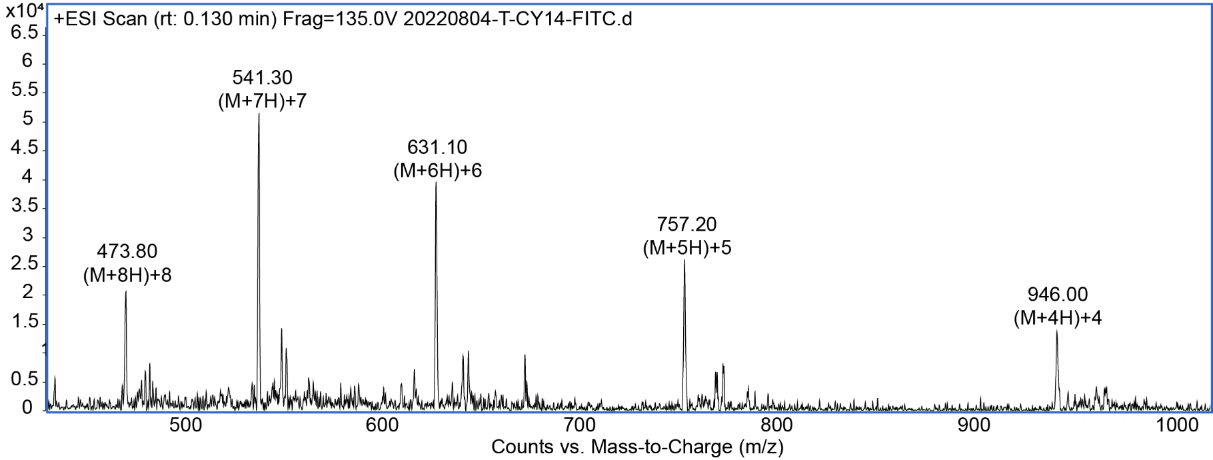


**Figure S32.** MS of compound FITC-T-CY14.


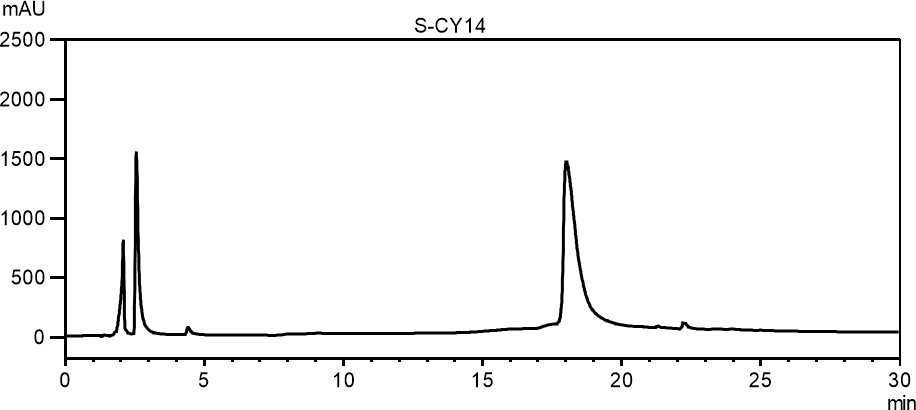


**Figure S33.** HPLC of compound S-CY14.


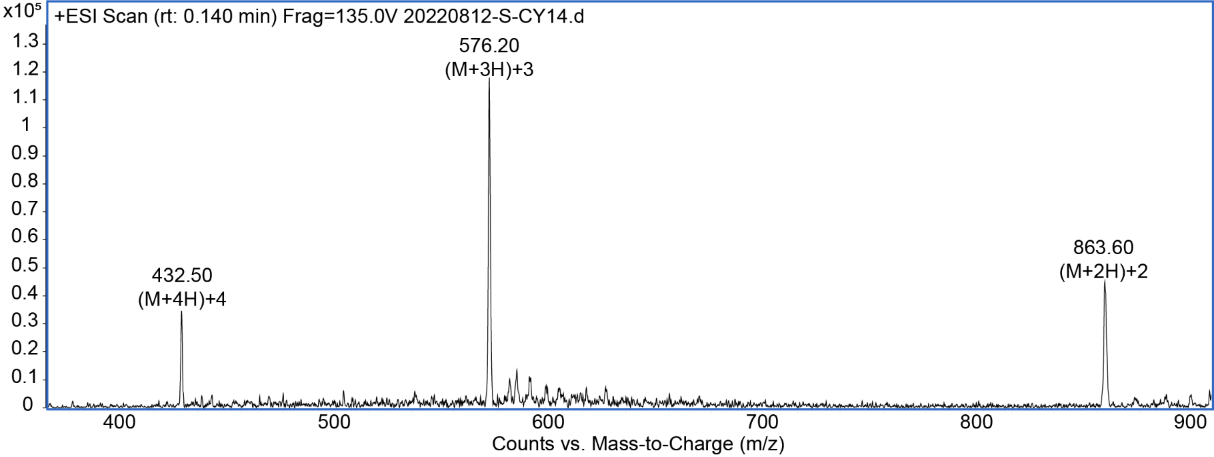


**Figure S34.** MS of compound S-CY14.


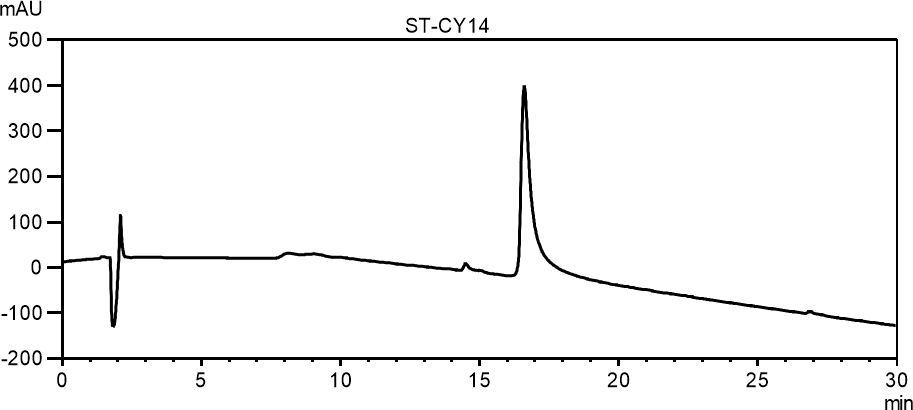


**Figure S35.** HPLC of compound ST-CY14.


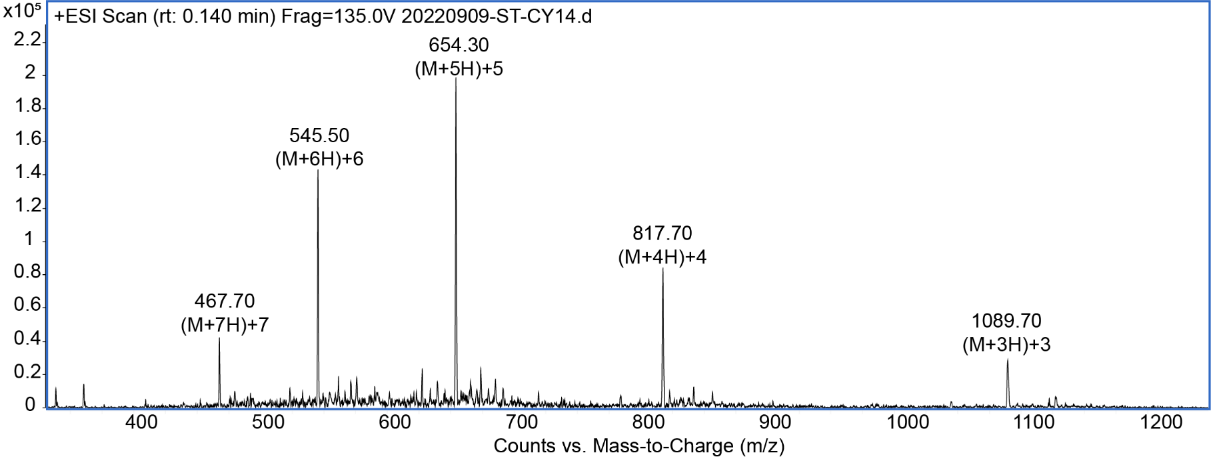


**Figure S36.** MS of compound ST-CY14.
